# Human salivary extracellular vesicles: A comparative study of isolation techniques

**DOI:** 10.1016/j.bpr.2026.100292

**Published:** 2026-10-09

**Authors:** Fanni Annamária Boros, Alexandra Gentschev, Jan Van Deun, Witold Szymanski, Johannes Graumann, Martin Regensburger, Philipp Arnold, Friederike Zunke

**Affiliations:** 1 Department of Molecular Neurology, University Hospital Erlangen, Friedrich-Alexander-University Erlangen-Nürnberg (FAU), Erlangen, Germany; 2 Department of Dermatology, University Hospital Erlangen, Friedrich-Alexander-University Erlangen-Nürnberg (FAU), Erlangen, Germany; 3 Institute & Core Facility of Translational Proteomics, Biochemical/Pharmacological Centre, Marburg University, Marburg, Germany; 4 Institute of Functional and Clinical Anatomy, Friedrich-Alexander-University Erlangen-Nürnberg (FAU), Erlangen, Germany; 5 Institute of Anatomy and Cell Biology, Otto-von-Guericke-Universität Magdeburg, Magdeburg, Germany

**Keywords:** extracellular vesicles, EVs, salivary EVs, size-exclusion chromatography, SEC, membrane affinity, MA, polymer precipitation, PP, nanoparticle tracking analysis, NTA, interferometric nanoparticle tracking analysis, iNTA

## Abstract

Saliva offers a valuable source of analyte for extracellular vesicle (EV)-based biomarker research. However, due to the complex composition of saliva, the isolation of salivary EVs remains technically challenging. The aim of our study is to systematically compare three salivary EV isolation techniques by assessing the obtained EV sample quality and quantity and to highlight differences between the three methods with respect to costs, isolation time, and potential for parallel sample handling (i.e., sample throughput). For the enrichment of EVs, we used three EV isolation techniques with saliva samples of four healthy donors: polymer precipitation, membrane affinity (MA), and size-exclusion chromatography. The vesicles obtained were characterized via transmission electron microscopy, SDS-PAGE and Western blot, and conventional nanoparticle tracking analysis (NTA) and interferometric nanoparticle tracking analysis (iNTA). Our results indicate that all isolation methods resulted in successful enrichment of EVs of similar size distribution. However, we observed differences with respect to vesicle yield and detectability of positive EV markers, potentially reflecting differences in vesicle integrity and sample purity. Assessment of particle number via NTA and iNTA approaches revealed prominent differences in EV number of MA samples but not in the case of samples prepared with other enrichment methods, highlighting the importance of the choice of analysis method. Our study highlights advantages and potential shortcomings of three salivary EV isolation approaches and calls attention to the importance of multimodal analysis of EV samples, combining approaches for EV marker detection, imaging, NTA, and iNTA.

## Why it matters

Extracellular vesicles (EVs) reflect the characteristics of cells from which they originate, and therefore are valuable sources of information on diseases affecting organs hardly or not at all available for sampling. Considering the noninvasive collection of saliva, salivary EVs are particularly promising candidates for deciphering disease-related processes and molecular signatures in a variety of disorders. In this study, we give a detailed description of three easy-to-use protocols for enrichment of human salivary EVs, and we assess these in terms of EV yield, purity, and vesicle integrity and evaluate the isolation methods concerning cost, hands-on time, and equipment requirements. We call attention to characteristics of these techniques, which can act as advantages and disadvantages depending on the research question concerning the obtained EVs.

## Introduction

Extracellular vesicles (EVs) are small, membrane-bound particles released by virtually all cell types. Based on their biogenesis, size, and functional characteristics, EVs are categorized into subtypes such as exosomes, macrovesicles, microparticles, and apoptotic bodies.^1^^,^^2^ EVs serve as means for cells to dispose of unwanted material, and additionally, they play an important role in intercellular communication by carrying biomolecules such as proteins, lipids, and nucleic acids.^3^^,^^4^^,^^5^ Importantly, the composition of both the cargo and membrane of EVs reflect their cellular origin,^6^ making EVs highly valuable as noninvasive diagnostic tools for insight into the physiological and pathological states of organs that are otherwise challenging to sample.^7^ Notably, EVs have been discussed to be able to cross biological barriers, such as the blood-brain barrier, further enhancing their potential in diagnostic applications.^8^^,^^9^ The diagnostic utility of EVs has been intensively explored in numerous disease contexts. These include malignant diseases^10^^,^^11^^,^^12^ and autoimmune diseases^13^ as well as neurodegenerative disorders such as Parkinson’s disease and Alzheimer’s disease.^14^ In these disorders, EVs provide critical information about cellular and organ health through their molecular cargo, including RNA species and protein biomarkers.^1^^,^^4^ The presence of EVs in a wide range of body fluids accessible in a noninvasive (such as urine and saliva) and minimally invasive (such as blood and cerebrospinal fluid [CSF]) manner has led to substantial interest in methods for EV isolation and characterization,^1^^,^^15^ yielding a wide variety of techniques.^1^^,^^16^

Of the various body fluids, saliva is particularly promising source of biomarkers due to its noninvasive collection and potential for providing insight into both systemic and localized conditions.^5^^,^^17^ It is a complex biofluid composed predominantly of water and comprising electrolytes, mucus, and antibacterial compounds as well as various enzymes. Its composition is influenced by age, health, and nutrition.^18^^,^^19^ The inherent complexity of saliva complicates sample processing and analysis, underscoring the importance of optimized methodologies for enriching salivary extracellular vesicles (salivary EVs).^5^^,^^17^^,^^20^ Although saliva is increasingly recognized as an easily, noninvasively accessible biofluid with diagnostic potential, systematic comparison of EV isolation methods from saliva has been limited. Here, we compare three methods, membrane affinity (MA), size-exclusion chromatography (SEC), and polymer precipitation (PP) (Figure 1), for the isolation of EVs from saliva samples. With our choices of these isolation techniques, we intended to compare methods which, while simple and doable in the absence of large equipment, result in preparations suitable for further downstream analyses in dedicated laboratories. To evaluate the quality of EV samples obtained via MA-, SEC-, and PP-based methods, following the recommendations of the MISEV guidelines,^1^ we use Western blot (WB), transmission electron microscopy (TEM), nanoparticle tracking analysis (NTA), and interferometric nanoparticle tracking analysis (iNTA) to assess EV yield, EV purity, and vesicle size distribution and integrity, and compare the isolation methods in terms of cost, hands-on time, and equipment requirements. Based on our results, we selected SEC to isolate salivary EVs in an independent sample set, for which we performed in-depth proteome analysis, revealing the presence of a range of positive EV markers in the samples and demonstrating the utility of the isolated EVs in such omics analyses.

**Figure 1:**
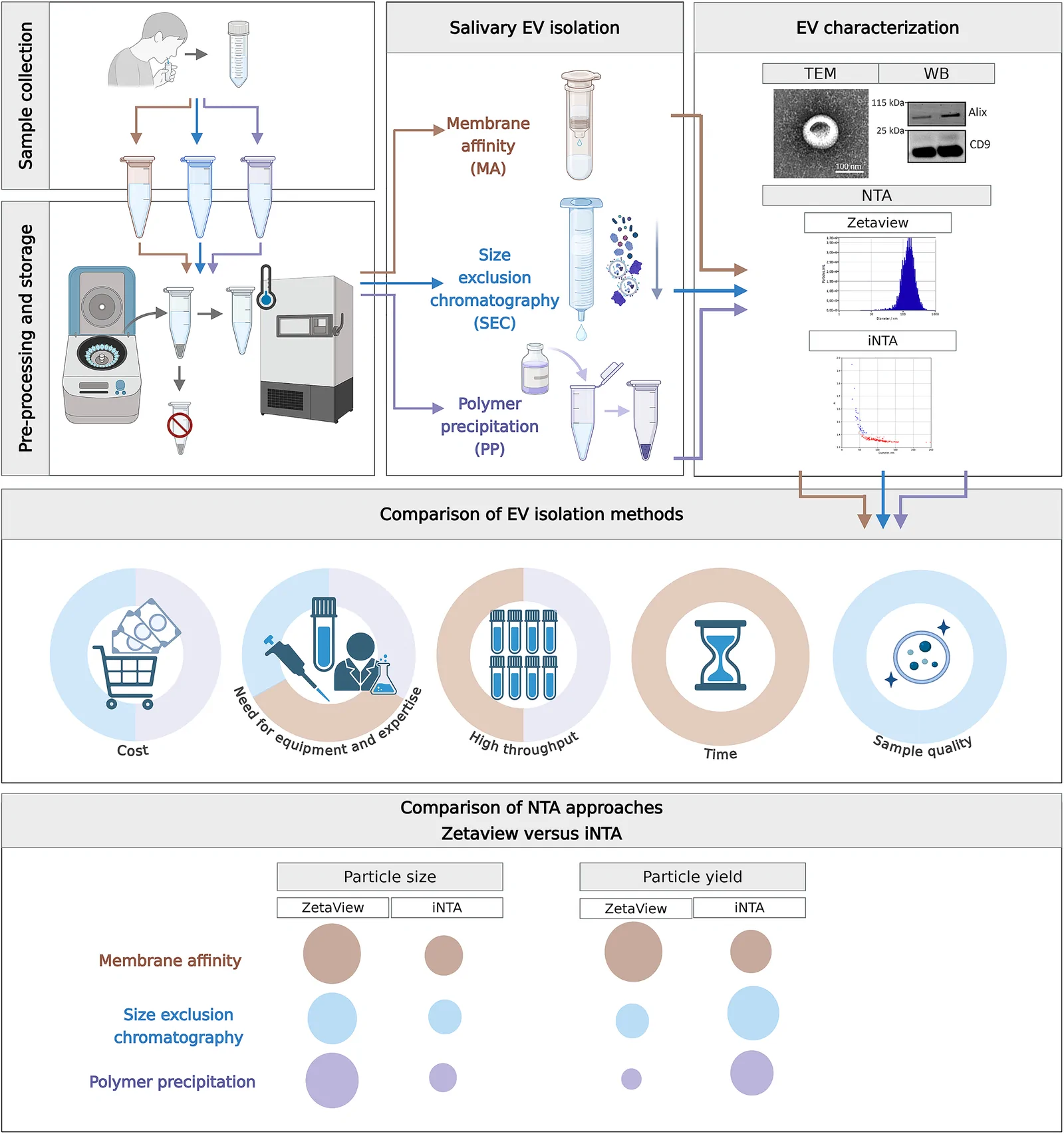
Study overview. Three salivary EV isolation techniques were evaluated for efficiency and performance by analyzing (1) EV markers and carryover proteins via Western blot (WB), (2) vesicle morphology and integrity by transmission electron microscopy (TEM), and (3) particle concentration and size with conventional nanoparticle tracking analysis (NTA) and its recent interferometric implementation (iNTA). While SEC and PP outperform the MA approach in terms of vesicle integrity, all methods present with an individual profile with respect to cost, experimentation time, sample throughput, and EV quality obtained.

## Materials and methods

### Study participants

Saliva samples were collected from four healthy volunteers. The donors included in the study ranged in age from 22 to 32 years and comprised two males and two females. For mass spectrometry (MS)-based shotgun proteomics analysis, an individual sample set consisting of salivary EV samples of six healthy donors was used. The male/female ratio of this study group was 2:4, with the age ranging from 42 to 78 years.

Collection, storage, and analysis of the samples was carried out following the approved ethical protocol #17-259_3-B. Written informed consent was obtained from all participants.

### Collection and storage of saliva samples

Sample collection was carried out following specific criteria. Participants were free of conditions or lesions affecting the oral cavity, such as infections, aphthae, or herpes, and had no autoimmune, premalignant, and malignant diseases. Participants were instructed to refrain from eating, drinking, smoking, or performing oral hygiene procedures for at least 1 h prior to sample collection, which took place between 9 a.m. and 2 p.m. For the collection of unstimulated saliva, participants were instructed to tilt their heads downward to allow saliva to pool naturally in the mouth. They then gently drooled or spat into a sterile 50-mL Falcon tube, avoiding coughing or forceful expectoration. From each participant, 4.5–7 mL of saliva was collected, which was placed immediately on ice and processed within 30 min after collection. To this end, 1.5-mL fractions of the collected samples were aliquoted in low-protein-binding microcentrifuge tubes (Sarstedt, cat. no. 72.706.600), and centrifuged at 3,000 × *g* for 20 min at 4°C to remove cells and large debris. The supernatant was carefully transferred into a new low-protein-binding tube, ensuring that the pellet remained undisturbed. To the aliquots, protease- and phosphatase-inhibitor cocktails (Protease inhibitor cocktail [PIC], Roche, cat. no. 11873580001, stock 25× and PhosStop [PS], Roche, cat. no. 04906837001, stock 20×, respectively) were added to a final concentration of 1×, and the aliquots were stored at −80°C until further use. Prior to EV isolation, the saliva samples were thawed on ice and centrifuged at 2,000 × *g* for 10 min at 4°C to remove any aggregates that may have formed during storage.

### Salivary EV isolation

For the isolation of salivary EVs, three different approaches were implemented: PP, an MA-based method, and separation via SEC. The former two methods include the use of commercially available kits, whereas SEC was performed with gravity-flow self-made columns. An overview of the methods is presented in Figure 2. The protocols are described in detail below.

**Figure 2:**
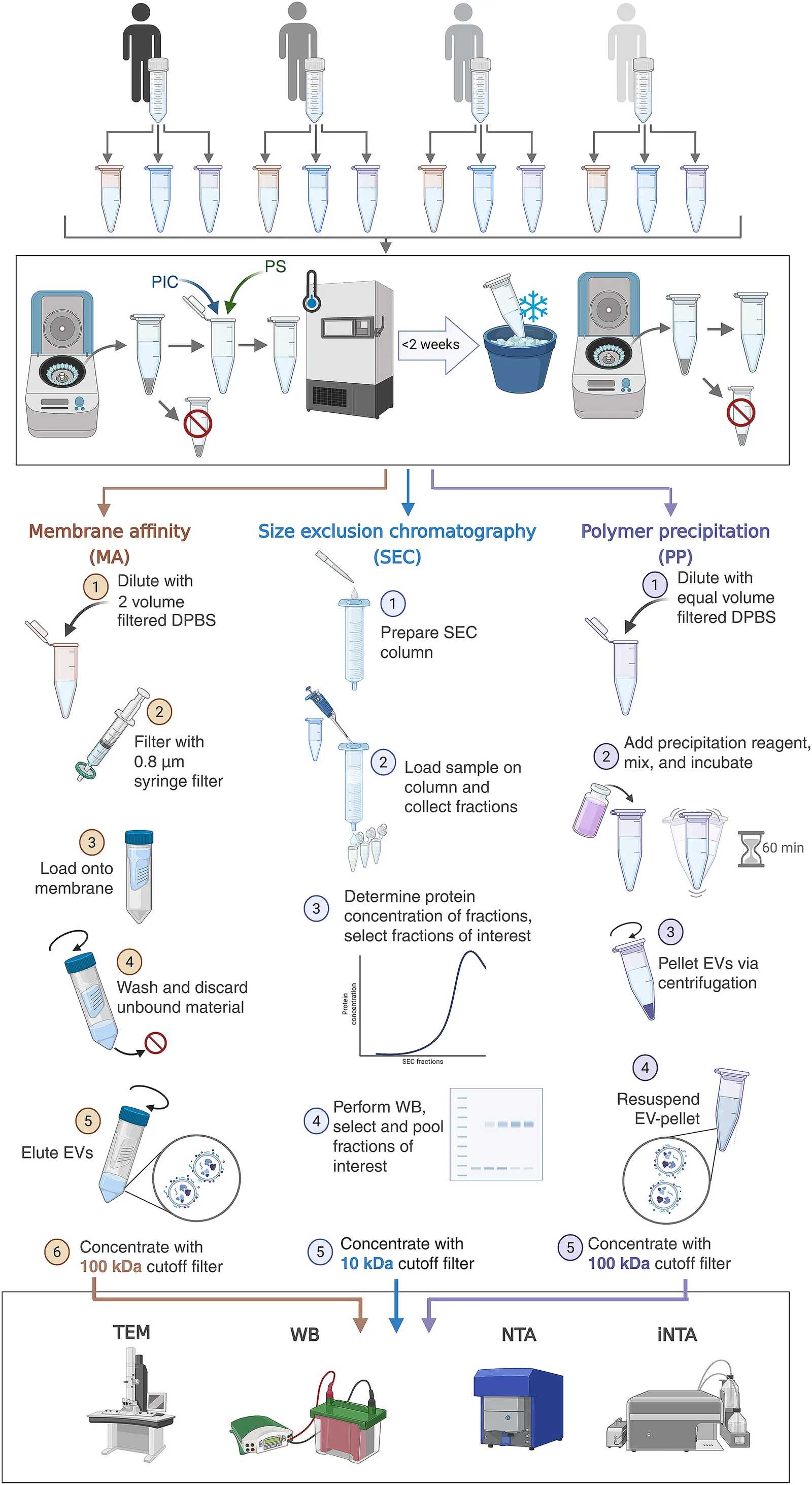
Overview of the utilized salivary EV isolation methods. Saliva was collected from four healthy donors and aliquoted in three vials. Following a centrifugation step and addition of proteinase and phosphatase inhibitor cocktails, the pretreated saliva samples were stored at −80°C until further use. On the day of EV isolation, the samples were thawed on ice and underwent a centrifugation step in order to remove possible aggregates formed during storage. One aliquot obtained from each donor was used in each EV isolation method. Salivary EV isolates prepared with membrane affinity (MA; brown accent color), size-exclusion chromatography (SEC; blue accent color), and polymer precipitation (PP; purple accent color) approaches were then analyzed with TEM, SDS-PAGE, and WB, NTA, and iNTA approaches.

#### MA-based method

For the MA-based EV isolation method, the commercially available ExoEasy Maxi Kit (Qiagen, cat. no./ID 76064) was used following the instructions of the supplier. The used buffer solutions were and are referred to here as described in the kit manual. 1.2 mL of saliva sample was diluted with 2 volumes of Dulbecco’s phosphate-buffered saline (DPBS; Sigma, cat. no. D8537-500ML) (DPBS used above and in all consecutive steps was 0.22-μm filtered), and the diluted sample was passed through a 0.8-μm-pore-size sterile syringe filter (Sartorius Minisart NML, cat.no.16592GUK). One volume of binding buffer (XBP) was added to equal volume of the filtered sample (3 mL), mixed gently by inverting the tube five times, and allowed to equilibrate to room temperature. The sample-buffer mixture was loaded onto an ExoEasy spin column and centrifuged at 500 × *g* for 1 min at room temperature. After discarding the flowthrough, 10 mL of wash buffer (XWP) was added to the column, followed by centrifugation at 3,000 × *g* for 5 min at room temperature. The flowthrough-containing molecules that were bound nonspecifically to the membrane were then discarded, and the spin column containing the membrane-bound EVs was transferred to a fresh collection tube. To elute the EVs, 900 μL of elution buffer (XE) was applied to the column membrane and incubated for 1 min at room temperature, and the column was centrifuged at 500 × *g* for 5 min to collect the eluted EVs. The elution step was repeated by reapplying the eluate to the membrane, followed by a 1-min incubation at room temperature and centrifugation at 4,000 × *g* for 5 min. The EV-containing flowthrough was transferred to a low-protein-binding microcentrifuge tube (Sarstedt, cat. no. 72.706.600), and the sample was supplemented with PIC (stock 25×) and PS (stock 20×), both at a final concentration of 1×.

#### SEC

##### Column preparation

For the isolation of salivary EVs via SEC, we followed a protocol adapted from Karttunen et al.^21^ and Böing et al.^22^ Sepharose CL-2B (Cytiva, cat. no. 17014001) was washed three times with DPBS of a volume equal to the set Sepharose volume, meanwhile allowing the washed matrix to reach room temperature. The SEC column was prepared using a 10-mL plastic syringe (BD Discardit II, cat. no. 309110). The plunger was removed, and an approximately 0.5 × 0.5-cm piece of nylon net filter of 20-μm pore size (Millipore, cat. no. 2002500) was placed at the syringe exit to prevent the Sepharose exiting the syringe. The syringe was filled with the washed Sepharose to create a column of height 6.5 cm. Once the stacked matrix reached the desired height, the exit of the syringe was closed (Braun Combi Stopper, cat. no. 4495152) leaving a layer of 0.5 cm DPBS on top of the column matrix. The top of the syringe was then covered with parafilm, and the column was left overnight at 4°C.

##### Sample loading and fraction collection

On the day of salivary EV isolation, the SEC columns were allowed to reach room temperature prior to sample loading.

Saliva samples were thawed on ice and centrifuged at 2,000 *×* g for 10 min at 4°C to remove aggregates formed during sample storage. 1.2 mL of saliva sample was carefully loaded onto the column, avoiding disturbing the even Sepharose layer. Once the whole sample volume was added, the collection of fractions of 500-μL volume started immediately. For each sample a total of 26 fractions were collected, which were immediately placed and kept on ice until consequent protein concentration measurement.

Following protein concentration measurement (see below), fractions preceding those that showed a prominent protein concentration increase were selected (typically fractions 7–12). Notably, in these fractions a small peak in protein concentration was observable, corresponding to the EV-rich fractions. The fractions of interest were further analyzed by SDS-PAGE and WB for EV-specific and carryover protein markers (see below) and, based on signal intensities, fractions were combined into a SEC_1_ and a SEC_2_ sample. For SEC_1_ samples, three fractions typically between fractions 7 and 10 were combined, as these showed high CD9 and low immunoglobulin G (IgG) signal. The consecutive, typically two fractions were combined for SEC_2_ samples, showing decreasing CD9 and increasing IgG content compared to SEC_1_ (Figure S1).

#### PP-based method

For EV isolation via PP, a commercially available precipitating reagent was used (Total Exosome Isolation Reagent, Thermo Fisher Scientific, cat. no. 4478360). Following thawing and centrifugation as described in “collection and storage of saliva samples,” a 1.2-mL saliva sample was diluted with an equal volume of DPBS, and the precipitating reagent was added, keeping a 1:5 final ratio to the volume of diluted saliva. The sample was mixed gently by inverting the tube ten times and incubated at 4°C for 1 h without any agitation. Following incubation, the mixture was centrifuged at 12,000 × *g* for 1 h 10 min at 4°C. The supernatant was carefully discarded, and the pellet containing the EVs was resuspended in 900 μL of DPBS supplemented with 1× PIC and 1× PS by gentle pipetting, followed by overnight incubation at 4°C to ensure that the pellet was fully dissolved (note that the EV pellet is often hardly or not at all visible).

### Sample concentration via ultrafiltration

Isolated EV samples were concentrated via ultrafiltration. In the case of the samples prepared by PP and MA, centrifugal filters of 100 kDa cutoff were used (Amicon Ultra 0.5-mL centrifugal filter device, cat. no. UFC510024) in order to decrease the amount of non-EV-related proteins in the samples. For SEC_1_ and SEC_2_ samples, filters of 10 kDa cutoff were used, as these samples contained fewer non-EV-related proteins, and the loss of EVs during ultrafiltration can be decreased by using filters of 10 kDa cutoff.^23^

Prior to use, the filter membrane was prerinsed by adding DPBS and centrifuging at 10,000 × *g* for 5 min at 4°C. After discarding the flowthrough, the filter was used within 20 min to prevent dehydration of and damage to the membrane. For the concentration process, EV-enriched samples were applied to the filter unit and centrifuged at 10,000 × *g* at 4°C until the sample volume was reduced to approximately 50 μL. To recover the concentrate, the filter unit was placed inverted in a microcentrifuge tube and centrifuged at 1,000 × *g* for 2 min at 4°C. The recovered sample was transferred to a low-protein-binding microcentrifuge tube and stored as 10-μL aliquots at −80°C until further analysis.

### Protein concentration determination

For determination of protein concentration of SEC fractions and concentrated EV samples, bicinchoninic acid (BCA) and micro-BCA (mBCA) assays were used, respectively. For the BCA assay (Pierce BCA Protein Assay Kit, Thermo Fisher Scientific, cat. no. 23225), the working reagent was prepared by mixing reagents A and B in 50:1 ratio, according to the manufacturer’s instructions. Standards were prepared by serially diluting bovine serum albumin (BSA) in DPBS to create solutions with a concentration ranging from 0.03125 mg/mL to 2 mg/mL. 10 μL of standards or samples (undiluted SEC fractions) were pipetted into a 96-well plate, 100 μL of the working reagent was added, and the plate was incubated at 37°C for 30 min. Absorbance was measured at 562 nm using a microplate reader (Clariostar, BMG Labtech), and protein concentrations were determined by comparing sample absorbance values to the standard curve.

For the mBCA assay (Pierce Micro BCA Protein Assay Kit, Thermo Fisher Scientific, cat. no.23235), the working reagent was prepared by mixing reagents A, B, and C in a 25:24:1 ratio as specified by the manufacturer. BSA standard was generated by the dilution of 2 mg/mL stock solution with DPBS to cover a 0.5–200 μg/mL concentration range. 150-μL aliquots of the standard were pipetted in duplicates into a 96-well plate. The EV-enriched samples were diluted 1:100 in DPBS in a final volume of 150 μL and added to the wells of the reaction plate in duplicates. 150 μL of the mBCA working reagent was added to each reaction, and the plate was covered and incubated at 37°C for 2 h. Absorbance was measured at 562 nm using a microplate reader (Clariostar, BMG Labtech), and protein concentrations were calculated against the standard curve.

### SDS-PAGE and WB

For SDS-PAGE and WB analysis, 10-μL SEC fractions or salivary EV samples containing 5 μg protein were mixed with sample loading buffer (NuPAGE 4× LDS sample buffer, Invitrogen, cat. no. NP0007) and sample reducing agent (NuPAGE 10× sample reducing agent, Invitrogen, cat. no. NP0004) and were heated at 70°C for 10 min. Samples were loaded onto precast Bis-Tris 4%–12% gradient gels (NuPAGE Mini Protein Gel, Bolt, 1 mm, Invitrogen, cat. no. NW04125BOX) set up in 1× running buffer (5 mM MES [2-(*N*-morpholino)ethanesulfonic acid], 5 mM Tris base, 0.01% SDS [sodium dodecyl sulfate], and 0.1 mM EDTA; pH 7,4) in a Mini Gel Tank (Invitrogen, cat. no. NW2000). For molecular weight marker, a prestained protein ladder (Thermo Fisher Scientific, PageRuler Plus prestained protein ladder, cat. no. 266190) was used. Electrophoresis was performed at 80 V for the initial 10 min, followed by 125 V for a total of approximately 80 min. Following SDS-PAGE, the proteins were transferred to a PVDF membrane (Immobilion-FL, Millipore, cat. no. IPFL00010, 0.45 μm pore size) in 1× transfer buffer containing 20% methanol in a mini blot module (Invitrogen, cat. no. NW2000) at constant 30 V for 1 h 10 min. Following transfer, the protein gel was stained with Coomassie brilliant blue (CBB; 0.02% CBB G-250, 5% aluminum sulfate hydrate, 10% ethanol, and 2% *ortho*-phosphoric acid) by gently rocking at room temperature overnight. The following day, CBB was discarded and replaced with Milli-Q water. The gel was kept hydrated in water for minimum 24 h until subsequent detection with an Odyssey M scanner (LI-COR, cat. no. 3350-00).

The membrane was fixed in 0.4% paraformaldehyde by rotating at room temperature for 20 min. The membrane was washed with Milli-Q water three times for 5 min each and stained with Ponceau S solution (Sigma-Aldrich, cat. no. P7170-1 L) by incubating at room temperature for 3–5 min until protein bands became visible. The dye was then discarded and the membrane rinsed with Milli-Q water prior to scanning with the Odyssey M scanner. Following scanning, the membrane was destained with transfer buffer and blocked in Intercept blocking buffer (LI-COR, cat. no. 927-60001) for 1 h, rotating at room temperature. Primary antibodies were added and incubated overnight at 4°C with constant rotation. As primary antibodies, CD9 (host species: rabbit, Cell Signaling Technology, cat. no. 13403) and Alix (host species: rabbit, Cell Signaling Technology, cat. no. 92880) were diluted 1:1,000 and 1:500 in Intercept Antibody Diluent (LI-COR, cat. no. 927-65001), respectively. The following day, the membrane was washed with 1× TBS-T (100 mM Tris [pH 7.4], 150 mM NaCl, and 0.1% Tween-20) three times for 10 min each. For detection, the membrane was incubated with a fluorescently labeled secondary antibody (IRDye 800CW donkey anti-rabbit IgG secondary antibody, LI-COR, cat. no. 926-32213) diluted 1:10 000 in TBS-T containing 2% fish gelatin for 1 h at room temperature with constant rotation under light protection. Finally, the membrane was washed again three times in TBS-T before imaging with the Odyssey M scanner. For the detection of human IgG, fluorescently labeled anti-human IgG antibody was used (Alexa Fluor 647 AffiniPure rabbit anti-human IgG [H + L], Jackson ImmunoResearch Laboratories, cat. no. AB_2339788) diluted 1:1,000 in TBS-T with 2% fish gelatin. Incubation and washing steps were identical to those for the secondary antibody described above. For signal detection, the iBright Imaging System (Thermo Fisher Scientific) was used. Images of whole blots and protein gels presented in this paper can be found in supplemental methods.

### NTA and iNTA

For the determination of particle concentration and size distribution, NTA was performed with two methodologies.

#### NTA with ZetaView instrument

The ZetaView (Particle Metrix, PMX-110) instrument was equipped with a CCD camera and a 405-nm laser. For calibration, standard dispersion of 100-nm polystyrene beads was used (Particle Metrix, cat. no. 700074) diluted 1:250,000 following the manufacturer's instructions. EV samples were diluted 250- to 4,000-fold with DPBS, and a final volume of 1 mL was injected into the instrument for measurement. Measurement settings used consistently for every sample were sensitivity 82, frame rate 30, and shutter 5.

#### iNTA

An iNTA measurement setup and analysis workflow was constructed and provided by the Max Planck Institute for the Science of Light as described previously.^24^^,^^25^ While nanoparticles are detected via dark-field microscopy in conventional NTA, their signal in iNTA results from the interference of their scattering with a reference laser beam, i.e., interferometric scattering (iSCAT) microscopy.^26^ This allows iNTA to be sensitive to smaller nanoparticles and decipher polydispersions more easily.

For measurements, passivated IBIDI μ-Slide 18-well chambered glass coverslips were used (Ibidi, cat. no. 81817). For passivation, mPEG2000-Silane (Laysan Bio) solution^27^ was added to the wells, allowed to dry, washed ten times with Milli-Q water, and allowed to air-dry in a chamber with silica beads until use. EV samples were diluted 50- to 400-fold in DPBS, and a 50-μL aliquot of each diluted sample was pipetted into a chamber. The microscope focus was adjusted to about 1 μm above the coverglass, and samples were recorded for 3 min at 10 kHz with a 50-μs exposure time. The field of view (FOV) was 6 × 6 μm. Particle size was derived from the combination of this information with the third root of the iSCAT contrast, which was calculated as a ratio of scattered field to reflected field.^24^ For distinguishing between EV and non-EV particles, a random forest classifier was used, as described previously.^27^ In brief, this classifier was trained on iNTA measurements of purified cell-culture EVs and purified lipoprotein particles. For each tracked particle, it returns the probability of this particle being an EV, based on its size, scattering cross-section, and refractive index (RI). Only particles with a higher than 80% probability of being an EV were included in the final analysis.

### TEM

The ultrastructural morphology of isolated salivary EVs was studied by TEM with a JEOL1400Plus TEM instrument as described previously.^28^^,^^29^ Samples were diluted 1:10 in DPBS. 3 μL of the diluted sample was applied onto a freshly glow-discharged (15 s at 25 mA) carbon-coated grid (300 mesh, Science Service Munich) and immediately washed twice with 5 μL of 2% uranyl acetate solution (aqueous). After air-drying for at least 60 s, samples were transferred into the TEM and imaged at 120 kV at 30.000× magnification.

### MS-based shotgun proteomics analysis

Isolated salivary EVs were lysed and processed using a modified single-pot, solid-phase-enhanced sample preparation (SP3) protocol.^30^ In brief, EV disruption and protein denaturation were achieved by adding 4% SDS in 50 mM TEAB in a 1:1 sample to buffer ratio, followed by heating at 95°C for 5 min with shaking. Due to low expected EV protein yields, protein quantification was omitted. Disulfide bonds were reduced and alkylated using 10 mM dithiothreitol at 90°C for 10 min and 15 mM iodoacetamide in darkness for 30 min, respectively. Protein cleanup was carried out on magnetic Sera-Mag carboxylate beads in 500-μL microcentrifuge tubes. Following ethanol and acetonitrile wash cycles, on-bead enzymatic digestion was performed using sequencing-grade trypsin/ at an estimated enzyme-to-protein ratio of 1:50 at 37°C overnight. A detailed protocol can be found in Szymanski and Graumann.^31^ Peptides were eluted in 35 μL of 0.1% formic acid supplemented with 0.015% (w/v) *n*-dodecyl-β-D-maltoside (DDM) and transferred to MS autosampler vials. Concentration was determined using the Pierce Fluorometric Peptide Assay (Thermo Scientific) and adjusted to a target concentration of 25 ng/μL prior to liquid chromatography(LC)-tandem MS analysis.

Peptide injection, separation, and measurement was performed as described previously.^32^ In brief, 50 ng of peptide mixture per sample was analyzed on a nanoElute nanoLC system coupled to a timsTOF Ultra mass spectrometer (Bruker Daltonics). Peptides were initially trapped on a PepMap C18 μ-Precolumn (5 mm, Thermo Scientific) and resolved across a PepSep Ultra C18 analytical column (75 μm × 25 cm, 1.5 μm particle size; Bruker Daltonics) at 300 nL/min for 45 min. Column effluent was ionized via a 20-μm CaptiveSpray emitter. Spectrum acquisition was performed in data-independent (dia-PASEF) mode applying the vendor’s standard parameters. Spectrum matching was performed in library-free mode using DIA-NN (v.2.2.0 Academia, Data-Independent Acquisition by Neural Networks)^33^ against the canonical human UniProtKB/Swiss-Prot database (reviewed November 2024, 20,429 entries). The search allowed fully tryptic cleavages (C-terminal K/R, omitting proline-adjacent sites) with up to one missed cleavage, peptide lengths of 7–35 residues, precursor charges from 1+ to 4+, and precursor/fragment mass ranges of 100–1,700 *m*/*z*. Fixed modifications were set to carbamidomethylation (C), while variable modifications included methionine oxidation (up to two per peptide) and initial methionine excision. Precursor false discovery rate was set at 1%, and match-between-runs (MBR) was applied. Downstream data processing and statistical analysis were carried out using the autonomics_1.17.16 package developed in-house (Bioconductor: https://doi.org/10.18129/B9.bioc.autonomics). Log_2_ intensities centered around sample means were used for quantitation and missing values imputed. Intensities where a protein group contained data from only two precursors (Np) per sample were converted to N/A (not applicable) for that particular sample. Proteins with a *q*-value of <0.01 were included for downstream analysis. Differential abundance of protein groups was evaluated using Bayesian moderated *t* testing as implemented by the limma^34^ package.

### Statistical analysis

Statistical analyses were performed with GraphPad Prism statistical software (GraphPad Prism v.9.5.1 for Windows, GraphPad Software, Boston, MA).

For testing of data distribution, the Shapiro-Wilk normality test was implemented. In the case of normal distribution, repeated-measures one-way ANOVA with Tukey's multiple-comparisons test or paired *t* test was used. If the normality test was not passed, the nonparametric Friedman test or Wilcoxon signed-rank test was implemented. *p*-values of less than 0.05 were considered statistically significant.

## Results

To evaluate the three methods (MA, SEC, and PP) for the isolation of EVs from saliva, the quality and quantity of the obtained EVs were assessed through a combination of TEM, WB, NTA, and iNTA, allowing for a comprehensive evaluation of particle yield, morphology, size distribution, presence of carryover proteins, and marker detectability. While all enrichment methods yielded vesicles, differences were observed with respect to the parameters mentioned above. In the case of SEC isolation protocol, both of the two separately collected combined SEC fractions were evaluated. Furthermore, we used SEC to isolate salivary EVs in an independent cohort of six donors to perform in-depth proteome analysis in order to assess the presence of a range of positive EV markers in the samples and the utility of the isolated EVs in MS-based proteomics analyses.

### Characterization of salivary EVs via TEM

EVs isolated using different methods from the four donors were analyzed by TEM to assess their morphology and integrity (Figure 3). Images of samples prepared using MA show fewer and smaller particles as compared to samples prepared with either of the other two methods (Figures 3 and S2). Furthermore, TEM images of MA samples showed noticeable background, apparently reflecting what can be observed in the input sample. EV samples isolated via SEC, especially in the earlier fractions (i.e., SEC_1_ samples) (Figure S1), exhibited well-defined and intact vesicles with minimal background contamination. The vesicles appeared consistently spherical and displayed clear membrane structure. While the images demonstrate that SEC_2_ samples (corresponding to fractions later than those referred to as SEC_1_) (Figure S1) also contain intact vesicles, a slight increase in background debris is noticeable as compared to SEC_1_ samples. As expected for a method separating particles based on their size, vesicles of SEC_2_ appeared to be smaller than those of SEC_1_.

**Figure 3:**
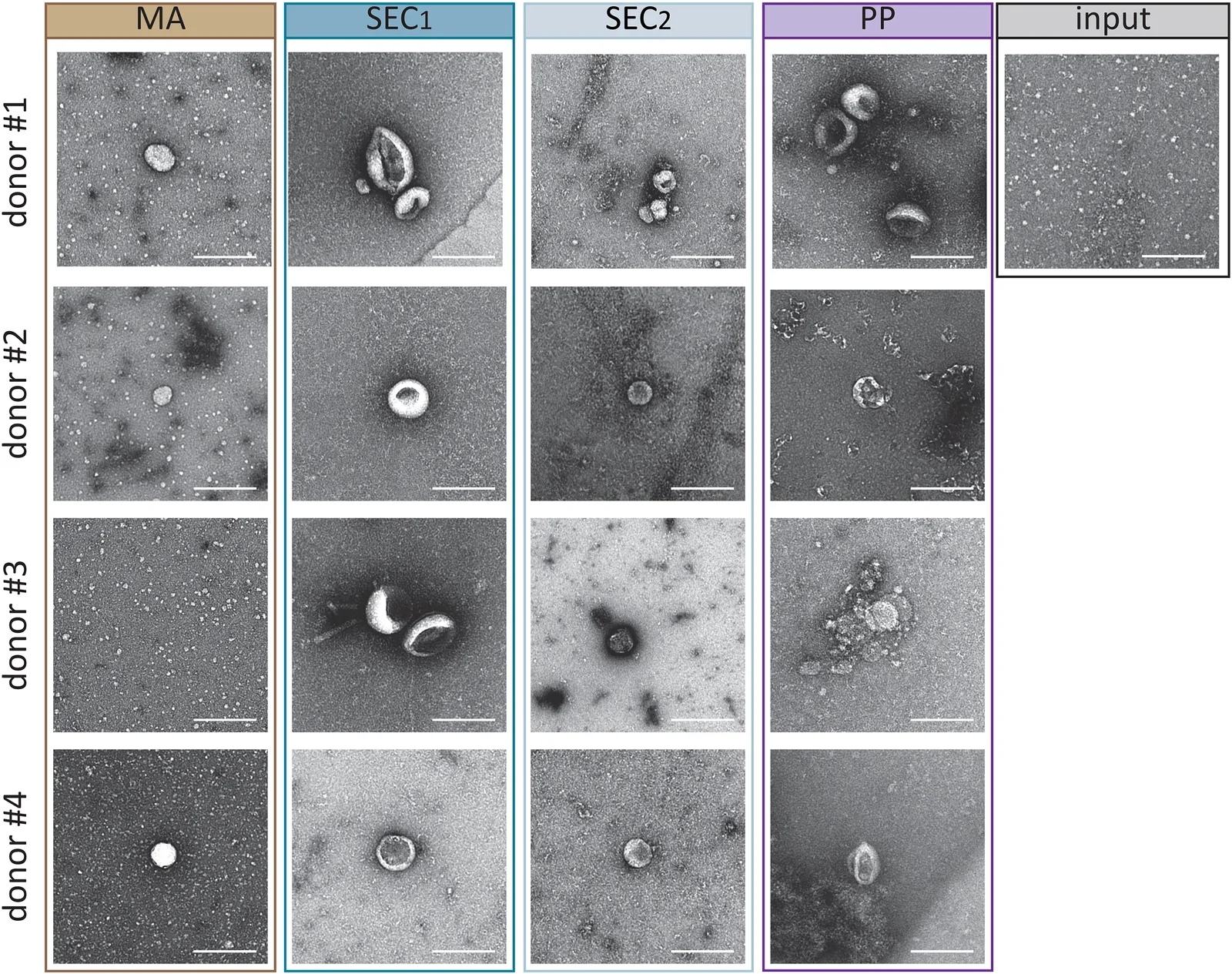
Representative TEM images of salivary EVs isolated with different methods. Images of samples prepared using MA method present with scarce, comparatively small vesicles. SEC results in the enrichment of well-defined, spherical EVs with minimal background. This is particularly true for early fractions (SEC_1_). In later fractions (SEC_2_), while intact vesicles are detectable, together with them a slight increase in the background is observable. Salivary EVs isolated using PP display noticeable aggregation and substantial background debris. A summary of TEM images of salivary EVs isolated with the different methods of samples of all donors (including the images here) can be found in Figure S2. MA, membrane affinity; SEC, size-exclusion chromatography; PP, polymer precipitation. Scale bars, 200 nm. Images of uncropped TEM images can be found in supplemental methods.

TEM images of samples prepared using PP to enrich salivary EVs display intact EVs with a cup-shaped morphology. Vesicles thus isolated, however, tend to form clusters and to aggregate (Figure 3).

Overall, TEM analysis suggests that of the three salivary EV-enrichment methods compared, SEC is most effective for achieving high purity and maintaining EV structural integrity. SEC yielded EVs with intact vesicle morphology and minimal contamination, rendering it a preferred choice for applications where low carryover protein presence and EV structural integrity are essential.

### Assessment of the presence of positive and negative EV markers using WB and MS-based shotgun proteomics analysis

To further evaluate the different EV isolation methods, SDS-PAGE and WB analyses were performed to detect EV-specific markers and non-EV-related proteins in salivary EVs prepared by MA, SEC, and PP (Figure 4). The analysis targeted Alix and CD9 as widely recognized positive EV markers, and IgG heavy chain and light chain (IgG hc and lc, respectively, in Figure 4) as frequent non-EV-specific carryover proteins, to assess EV enrichment and sample quality.

**Figure 4:**
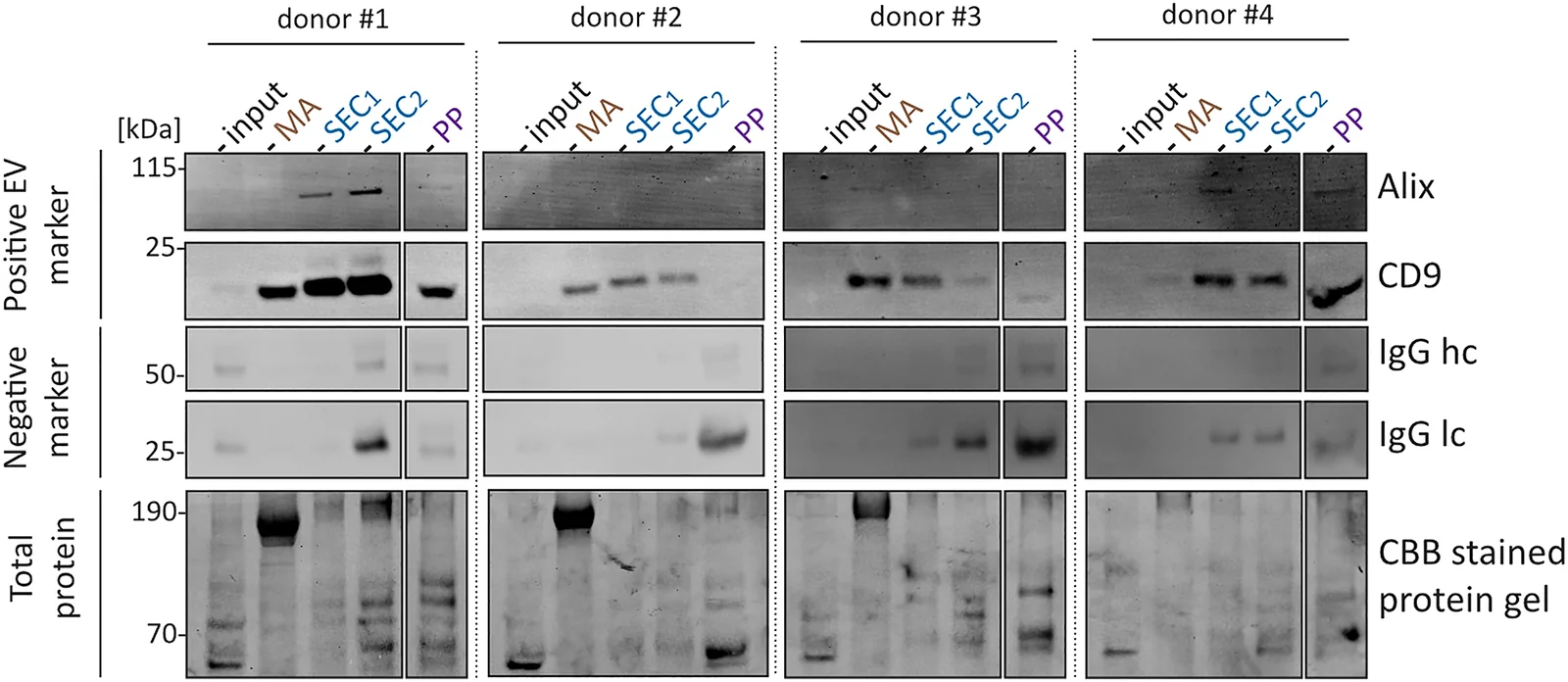
SDS-PAGE and Western blot analyses of salivary EV samples obtained by the compared three isolation methods. 5 μg protein samples prepared by the method indicated on top (MA, membrane affinity; SEC_1_ and SEC_2_, size-exclusion chromatography early and later fractions, respectively; PP, polymer precipitation) and input samples (i.e., saliva sample thawed on ice, centrifuged at 2,000 × *g* for 10 min at 4°C, and the supernatant collected) were separated on 4%–12% SDS-PAGE. Proteins were blotted and probed with the antibodies indicated on the right, and protein retained in the gel matrix after blotting was stained with Coomassie brilliant blue (CBB) to visualize the total protein pattern. Images of whole blots and protein gels can be found in supplemental methods.

CD9 is a member of the tetraspanin family.^35^ It is a glycoprotein that structurally consists of four transmembrane domains and is mainly present in the plasma membrane.^36^^,^^37^ In contrast, Alix is a cytosolic protein, first described as an interactor of the calcium-binding protein ALG-2 and later recognized as a regulator of the endolysosomal system.^38^ Its localization is intravesicular, and therefore its presence in EV preparations may be considered as an indicator of maintained vesicle integrity during the EV isolation process.

Saliva is a complex biofluid and a component of the first defense line in the oral cavity, a gateway to the body. Acting as an immune barrier, it offers first-line protection against potentially pathogenic microorganisms.^19^ To this end, saliva is rich in molecules participating in the immune response, such as immunoglobulins.^39^ In this study, we used IgG as a marker for the presence of carryover proteins co-isolating with the EVs in preparations, and thus for evaluation of sample purity. Given the abundance of carryover proteins, corresponding differences between preparations may be evaluated by methods as unspecific as CBB staining of protein gels (Figure 4).

In salivary EV samples prepared via MA, the lack of IgG signal and the faint apparent global protein signal visible by CBB indicate carryover proteins to be present at low abundance. CD9 signal was consistently detected across all donors, while Alix was barely detectable or absent. The low abundance of the latter indicates compromised vesicle integrity, resulting in partial loss of intravesicular content throughout the isolation procedure (Figure 4).

Similarly, in salivary EV samples isolated using SEC (SEC_1_ samples), the protein signal on CBB-stained protein gels and IgG heavy- and light-chain signals indicate low amounts of background protein. A comparison of WB images of early and later SEC fractions (SEC_1_ versus SEC_2_) demonstrates the positive EV marker CD9 to be consistently present in SEC_1_ samples and Alix signal also to be detectable in half of the SEC_1_ samples. Alix is detectable in the SEC_2_ sample of only one donor, and in the majority of these samples a more prominent non-EV-specific IgG contamination is present as compared to SEC_1_ samples. These results indicate that SEC is suitable for separating EV- and protein-rich fractions (Figure 4). The protein signal visible on CBB-stained protein gels of samples prepared with the PP method presents the closest resemblance to that of the input saliva samples, and the IgG heavy-chain signal detected across all donors further supports the notion of the presence of residual proteins. The detectability and/or signal strength of the positive EV markers CD9 and Alix showed great variability across PP samples of the different donors (Figure 4).

Following on from our observations based on TEM and WB analysis, i.e., maintained vesicle integrity, the presence of low amount of carryover proteins, and the detectability of CD9 and Alix in SEC isolated samples, we prepared salivary EVs via SEC in an independent sample cohort of six donors and performed MS-based shotgun proteomics analysis of SEC_1_ samples to further validate successful isolation of salivary EVs via the method (Figure S3). The results indicate the presence of all selected positive EV markers (FLOT1, CD63, ALIX, CD9, CD81, and TSG101), with comparable peptide coverage and protein abundance profiles across the analyzed samples (Figure S3).

### Characterization of salivary EVs via two NTA approaches

To compare the isolation protocols in respect of salivary EV yield and size, NTA and iNTA were performed with two instruments: the commercially available ZetaView (Particle Metrix) and the iNTA setup (Max Planck Institute for the Science of Light). The latter has a higher sensitivity for smaller particles and allows the distinction of EVs from non-EV particles by measuring the RI.^24^^,^^25^^,^^27^ Besides using the instruments for comparing the effectiveness of the EV isolation methods by determining particle yield and particle size, we also analyzed the comparability of the readouts obtained by NTA and iNTA instruments (Figure 5).

**Figure 5:**
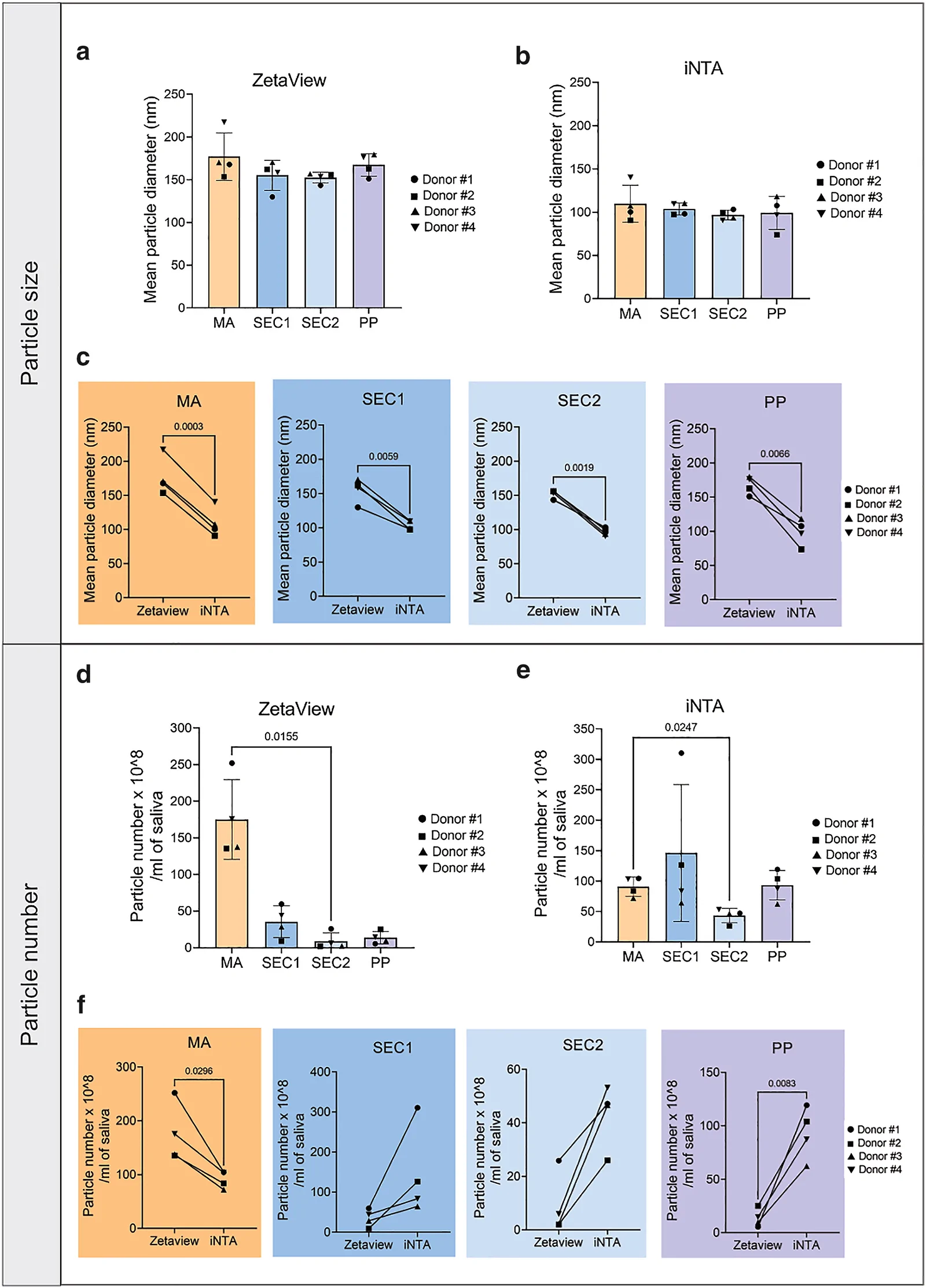
Comparison of isolated salivary EVs obtained by different isolation methods in respect of particle sizes and abundances as determined by ZetaView and iNTA. (a and b) Mean particle diameter (nm) measured by ZetaView (a) and iNTA (b) show no significant differences when comparing samples prepared with the different isolation methods. (c) Direct comparison of mean particle diameter between ZetaView and iNTA for each isolate reveal significantly smaller diameters when measured with iNTA. (d and e) Particle concentration (×10^8^ particles/mL saliva) measured by ZetaView (d) and iNTA (e) show significant difference between MA and SEC_2_ samples. (f) Comparisons of particle numbers of the same preparations measured with ZetaView and iNTA reveal method-specific differences: significantly higher particle counts in PP samples and significantly lower particle counts in MA samples are observable when measured by iNTA compared to results obtained with ZetaView. Each data point represents an individual sample. Each bar in graphs of panels (a, b) and (d, e) represents the mean of the four data points, and error bars represent ± SD.

Analysis of particle size and yield across the different EV isolation methods revealed method-dependent differences. ZetaView measurements of mean particle diameter showed relatively consistent values across all isolation techniques, with average sizes ranging from approximately 150 nm to 180 nm (Figure 5a). Similarly, iNTA measurements indicated consistent mean particle diameters across all methods, although the detected sizes were generally smaller, ranging from approximately 90 nm to 120 nm (Figure 5b).

Statistical analysis revealed no significant size differences of particles isolated by the different methods irrespective of the NTA instrument used, indicating that all EV isolation techniques resulted in the enrichment of EVs of the same-sized population.

However, a direct comparison of the NTA and iNTA platforms showed significant differences in measured particle diameter for each of the isolation techniques (Figure 5c). In every case, iNTA results show significantly smaller particle sizes compared to ZetaView. This systematic bias is related to the intrinsic nature of the signals in NTA and iNTA. In the former method, particles are detected via their scattered intensity in a dark-field configuration. This makes the signal proportional to the second power of the particle polarizability (*α*^2^) and, thus, sixth power of the particle diameter *D*^6^. The iSCAT signal used in iNTA, on the other hand, is proportional to the scattered field and therefore scales as *α*^3^, or correspondingly as *D*^3^.^26^ In a nutshell, the stronger size dependence of NTA skews the detection toward larger particles.

With respect to particle yield, ZetaView analysis revealed significant differences between methods (Figure 5d). The MA method produced the highest apparent particle yield across all donors, while SEC_2_ samples consistently returned the lowest yield (Figure 5d). A significant difference in particle size of MA and SEC_2_ samples was also reflected in the results of iNTA analysis (Figure 5e). However, the particle number of MA samples as determined by iNTA did not show such a stark difference in comparison to the other isolation methods as was seen when measured with NTA (ZetaView) (Figures 5d and 5e).

Interestingly, a comparison of particle counts between the two platforms showed that iNTA detected higher numbers of particles in SEC_1_, SEC_2_, and PP samples as compared to ZetaView. In the case of PP, the difference reached statistical significance (Figure 5f). Conversely, for MA samples, iNTA reported significantly lower particle numbers compared ZetaView measurements. When implementing the EV classifier for iNTA data (Figure 6a), we found that the average percentage of EV particles across all isolation methods showed similar values (Figure 6b). Similarly, the EV particle per protein ratio showed no significant difference between the sample groups prepared with the different isolation techniques (Figure 6c).

**Figure 6:**
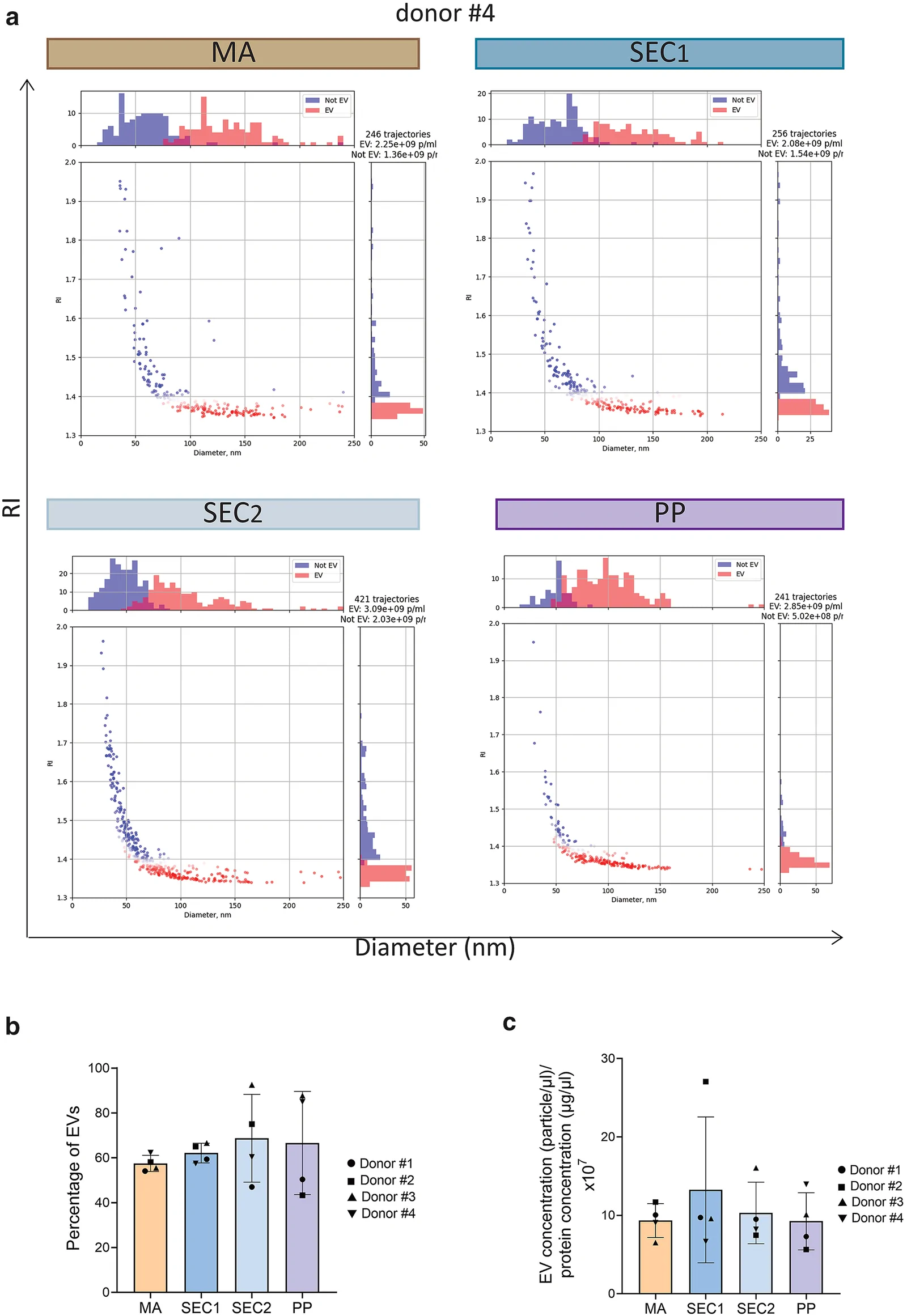
Determining EV and non-EV subpopulations with iNTA. (a) Representative graphs of particle-size distributions in EV isolates obtained from samples of the same donor with the use of different isolation methods. The plots show particle diameter (nm) plotted against refractive index (RI). Particles with a size-RI profile consistent with EVs are represented as red data points, and particles classified as non-EVs are depicted in blue. (b) All isolation methods yielded similar results in terms of the percentage of EV particles. (c) Similarly, no significant difference was observed between the isolation methods when comparing EV particles (determined by implementing the EV classifier for iNTA data) per protein ratio of the samples. In panels (b) and (c) each data point represents an individual sample, each bar shows the mean of the four data points, and error bars represent ± SD.

Graphs of particle diameter plotted against the third root of iSCAT contrast corresponding to the data shown in Figure 6a can be found in Figure S4.

### Comparison of MA, SEC, and PP salivary EV isolation methods regarding practical aspects

Due to the complex composition of the biofluid, the isolation of EVs from saliva presents challenges. To ensure reliable future diagnostic applications, it is essential to identify the isolation method that best suits downstream applications in terms of EV quality and quantity.

In addition to the differences described above concerning sample purity and EV yield, an evaluation of the three tested isolation methods revealed notable differences in cost, required equipment, throughput, and experimental workload (Table 1). Of the compared methods, MA was the most expensive but required no specialized equipment and allowed for high-throughput processing. With a sample handling time of only 20 min, it was the most time efficient of the methods compared. SEC using columns packed in-house represented the most labor-intensive and time-consuming option. While it costs less than MA, high-throughput parallelization was not feasible, and approximately 3.5 h of hands-on time was required per sample. Including additionally required incubation (to allow the column matrix to fully set and equilibrate with buffer and in temperature, time for running and staining-destaining PAGE, and time required for preparing WBs), the overall experimental time exceeds that of the other methods tested. The consistent EV quality and low amount of carryover proteins demonstrated by the results of TEM and WB analysis (Figures 3 and 4), however, may render its use justified for applications requiring stringent sample purity and integrity. The PP method, using a commercial reagent, was similarly cost effective (though highly dependent on sample volume utilized), required no specialized equipment, and enabled the parallel processing of multiple samples. However, it implies an overnight incubation step, resulting in a total isolation time exceeding that of the MA approach.

**Table 1:** Comparison of salivary EV isolation methods in respect of cost, sample handling, and experimental time requirements

Method	Costs and sample handling			Experimental time/sample (approximately)	
	Reagent cost per isolation (approximately)	Need for special laboratory equipment	Processing of large number of samples simultaneously	Hands-on time	Total time
Membrane-affinity	€ €	no	yes	20 min	20 min
SEC	€	no	no^a^	3.5 h	10 h + 2× overnight incubation/waiting time^b^
Polymer precipitation	€ (highly dependent on starting sample volume)	no	yes	20 min	2.5 h+ overnight incubation

Cost estimates are based on sample processing and reagent procurement on standard laboratory scale. Hands-on time requirement estimation is based on processing samples by a single trained individual from laboratory personnel. SEC, size-exclusion chromatography

a With further process optimization medium throughput achievable.

b Including column preparation and WB analysis for fraction selection.

## Discussion

This study aimed to evaluate and compare various salivary EV isolation methods, assessing their effectiveness in minimizing carryover protein amount while preserving EV integrity and yield. Our primary goal was to identify a cost-effective method that results in EV preparations suitable for downstream analyses while easily performable in a routine laboratory. We tested three EV enrichment protocols based on PP, MA, and SEC, and we assessed the quality and quantity of enriched EVs via TEM, WB, and NTA analysis with two modalities. Published salivary EV isolation methods focus primarily on ultracentrifugation-based approaches and techniques using magnetic bead immunocapture.^5^^,^^40^^,^^41^ To the best of our knowledge, this is the first study to compare PP, MA, and SEC for salivary EV isolation side by side and to compare results of NTA (ZetaView) and iNTA when analyzing the obtained salivary EVs.

Our comparative analysis of EV isolation methods highlights important differences in sample quality and quantity (Table 2). According to results of ZetaView analysis, the MA approach resulted in samples with the highest particle yield. This however, is in stark contrast to the results of iNTA analysis and to that observed in TEM images (Figure 3), which present hardly any intact vesicles. The notion of compromised vesicle integrity in MA sample preparations is supported by the absence of the intravesicular marker Alix in MA samples (Figure 4) and by iNTA results showing that MA samples contain a slightly lower percentage of EVs among all detected particles as compared to the other two methods (SEC and PP) (Table 2). A further explanation for the discrepancy between TEM and iNTA versus NTA results could be in an unknown component of the elution buffer used for the MA protocol. While both analysis methods that require the fixation and attachment of the vesicles to a surface, such as TEM and iNTA, yielded low EV count, with the use of NTA method, for which the particles remained dispersed in a solution, larger EV numbers were detected. This notion thus might point to the interdependence between the method of EV isolation used and the choice of analytical approach.

**Table 2:** Comparison of EV sample quality and quantity across three salivary EV isolation methods

Method	EV sample quality and quantity							
	Presence of intact vesicles (based on [1] TEM results and [2] presence of Alix determined via WB)	Presence of carryover proteins (based on [1] CBB staining of protein gel and [2] WB results of IgG detection)	EV particle- and protein-concentration ratio (particle count × 10^7^/μg protein) (determined by iNTA and mBCA; mean ± SD)	Particle size (mean diameter in nm; mean ± SD)		Particle yield (particle number × 10^8^/mL saliva; mean ± SD)		Percentage of EVs of total particle number (determined by iNTA; mean ± SD)
				NTA (ZetaView)	iNTA	NTA (ZetaView)	iNTA	
Membrane-affinity	concerning/questionable	considerable amount of carryover proteins	9.3 ± 2.2	181.1 ± 27.5	109.7 ± 21.5	179.3 ± 51.2	90.8 ± 15.8	57.5 ± 3.6
SEC	good	good separation of EV- and protein-rich fractions	13.3 ± 9.3	155.3 ± 17.6	103.8 ± 7.1	35.4 ± 21.6	146.3 ± 112.6	62.1 ± 4.4
Polymer precipitation	sufficient	considerable amount of carryover proteins	9.2 ± 3.6	167.4 ± 13.2	99.1 ± 19	13.7 ± 8.5	93.3 ± 24.4	66.6 ± 23

Results on EV quality and quantity are compared based on values determined by the methods indicated in the first column. SEC (SEC_1_) samples contained intact vesicles and efficient separation of those from carryover proteins. TEM and WB analysis of MA samples suggest high number of broken and fragmented vesicles and substantial amount of carryover proteins. Samples prepared with PP contain intact vesicles often displaying the typical cup-shaped morphology in TEM images, and the EVs often appear in clusters. EV-particle number/protein ratios (particle counts as determined by implementing the EV classifier for iNTA data per μg protein) do not differ significantly between the samples prepared with the different isolation methods; nonetheless, salivary EV samples isolated with SEC show the highest value, suggesting the lowest amounts of carryover proteins per EVs in these samples. The largest average particle size was measured in MA samples both via ZetaView and iNTA measurements; however, in terms of particle yield, the two modalities were not in accord. According to ZetaView measurement data, MA samples demonstrated the highest particle yield, whereas according to iNTA data, SEC seemed to be the most effective isolation approach. Results of iNTA, furthermore, showed that PP samples showed the highest percentage of EVs relative to total particle number.

SEC, size-exclusion chromatography; TEM, transmission electron microscopy; WB, Western blot; CBB, Coomassie brilliant blue; EV, extracellular vesicle; mBCA, micro-bicinchoninic acid (assay); NTA, nanoparticle tracking analysis; iNTA, interferometric nanoparticle tracking analysis.

SEC-based EV isolation presented the most effective separation of EVs from carryover proteins while maintaining vesicle integrity (Figures 3 and 4). SEC also yielded a relatively high apparent percentage of EVs within the total particle population, although the absolute particle yield was inferior to that of the other methods (Table 2).

The PP approach resulted in the samples with the comparatively lowest particle yield as measured with ZetaView, while the particle number was comparable to that in samples prepared using MA when assessed with iNTA (Table 2). With respect to the protein content of the samples, the detectability of CD9 was inconsistent throughout the four samples. The total protein signal in the PP samples resembled most closely that of the input saliva as compared to the companion isolation methods (Figure 4, CBB-stained protein gels). TEM analysis revealed vesicles of cup-shaped morphology, often presenting in clusters, indicating EV aggregation. The observed EV aggregates are in line with findings of TEM analysis of plasma EV samples enriched via polymer precipitation.^42^^,^^43^ The presence of these aggregates could lead to inhomogeneity within the samples and contribute to the observed inconsistency of our WB results. According to iNTA measurement results, PP samples showed the highest percentage of EVs among total particles among the EV isolates prepared by the three tested methods; however, the high standard deviation within the group further supports the notion of inconsistent behavior of the method (Table 2).

Although the small sample size limits the generalization of conclusions and should be taken into account when interpreting the results, we feel that our data highlight characteristics of the different approaches which, depending on the scientific questions and downstream EV analyses, may represent advantages or disadvantages.

This is exemplified by the findings for SEC EV samples. In line with the findings of reports on blood-derived EVs isolated with SEC,^44^^,^^45^ our TEM and WB results indicate samples of relatively low carryover protein content as well as maintained EV morphology and integrity, rendering SEC a preferable choice for applications where low non-EV protein content and intact EV structure are essential. A PP-based technique may, however, be preferable in the context of applications where these specific characteristics are less critical, and high EV yield and high-throughput sample processing are paramount. These results call attention to the fact that there is no one-size-fits-all isolation method for saliva EVs, as the method of choice highly depends on costs and personnel and equipment availability, as well as targeted sample quality and quantity. Independent of the chosen isolation technique, it is crucial to characterize the obtained EV samples using a multimodal approach employing divergent techniques to assess the presence of EV- and non-EV-related proteins as well as EV morphology and abundance.

A particular critical aspect of EV characterization in this context is the accurate quantification of particle size and concentration, typically achieved via NTA. As shown previously for plasma-derived EV samples, conventional NTA is not able to discriminate between different particle types that have similar size. Furthermore, its sensitivity is limited for particles below ∼50–60 nm, and sample polydispersity or aggregation can further complicate accurate sizing.^46^ Together, these factors result in an inaccurate picture when looking at size-based analysis alone. In contrast, iNTA includes RI as a physical parameter, which allows to distinguish EVs from other particles. In addition, it is able to estimate overall particle concentration more accurately than conventional NTA by counting trajectories crossing the focal plane rather than in a field of view of ill-defined dimensions.^25^ The added value of iNTA is especially clear for the MA sample, which was found to contain a large number of particles but few EVs.

These findings also underscore the importance of cross-validation with orthogonal techniques, such as TEM or single-particle interferometric reflectance imaging (SP-IRIS), and highlight the need for standardization in EV quantification protocols.

## Conclusion

In conclusion, this study provides protocols for three different salivary EV isolation approaches and presents a comprehensive comparison of EVs obtained using them. Our findings highlight both methods of isolation and analysis to significantly impact the observed characteristics of salivary EV preparations, warranting particular care in inter-lab transfer and modification of established EV isolation protocols.

## Data and code availability

The data sets used and/or analyzed during the current study, including R code used for data processing and statistical analysis alongside the MS raw data, are available from the corresponding authors on reasonable request.

## Acknowledgments

We thank Anna Kashkanova, Alexey Shkarin, Tobias Utikal, and Vahid Sandoghdar from the Max Planck Institute for the Science of Light for constructing and providing the iNTA instrument. Furthermore, we would like to thank all donors of this study, as well as Dejana Taneva Hristov and Zhanetta Zhatarova for excellent technical assistance. Figures 1 and 2 were created with BioRender.com. This work is supported by the Interdisciplinary Center for Clinical Research (Interdisziplinäres Zentrum für Klinische Forschung; IZKF, Erlangen) with a Junior Research Program J109 to F.A.B., a SYNERGY grant S3 (AstroFINDER) to F.Z., and SYNERGY grant S4 (DISCOVER) to J.V.D. and P.A. Further support was granted through funding by the 10.13039/100000864Michael J. Fox Foundation (MJFF-021325) to P.A. and F.Z., as well as by the 10.13039/501100001659Deutsche Forschungsgemeinschaft (DFG, German Research Foundation) – 505539112 – KFO 5024 (A02) to F.Z. M.R. was supported by the 10.13039/501100002347Federal Ministry of Research, Technology and Space (ACS_iIMMUNE, 01E02105).

## Author contributions

F.A.B. and A.G., data acquisition, analysis, and interpretation, and writing the initial manuscript; F.A.B., study design, figures, and acquisition of funding, revision; M.R., resources (biosamples) and donor recruitment; J.V.D., methodology (iNTA); W.S. and J.G., data acquisition, analysis, and interpretation (proteomics); P.A., acquisition and interpretation of data (TEM) and acquisition of funding; and F.Z., study design, revision, interpretation, and supervision, acquisition of funding, and writing the initial manuscript. All authors agree to the revision of the final manuscript.

## Declaration of interests

The present work was performed in (partial) fulfillment of the requirements for obtaining the degree Dr. med. dent. (A.G.).

## Supplemental information

Document S1. Figures S1–S4 and supplemental methods

Document S2. Article plus supplemental information
