# Supplementary material for: Human salivary extracellular vesicles: A comparative study of isolation techniques
Source: Biophys Rep (N Y). 2026 Oct 9;6(4):100292. doi: 10.1016/j.bpr.2026.100292 (PMC13721098; doi:10.1016/j.bpr.2026.100292)

Biophysical Reports, Volume *6*

Supplemental information

Human salivary extracellular vesicles:

A comparative study of isolation techniques

Fanni Annamária Boros, Alexandra Gentschev, Jan Van Deun, Witold Szymanski, Johannes Graumann, Martin Regensburger, Philipp Arnold, and Friederike Zunke

Supplementary Material

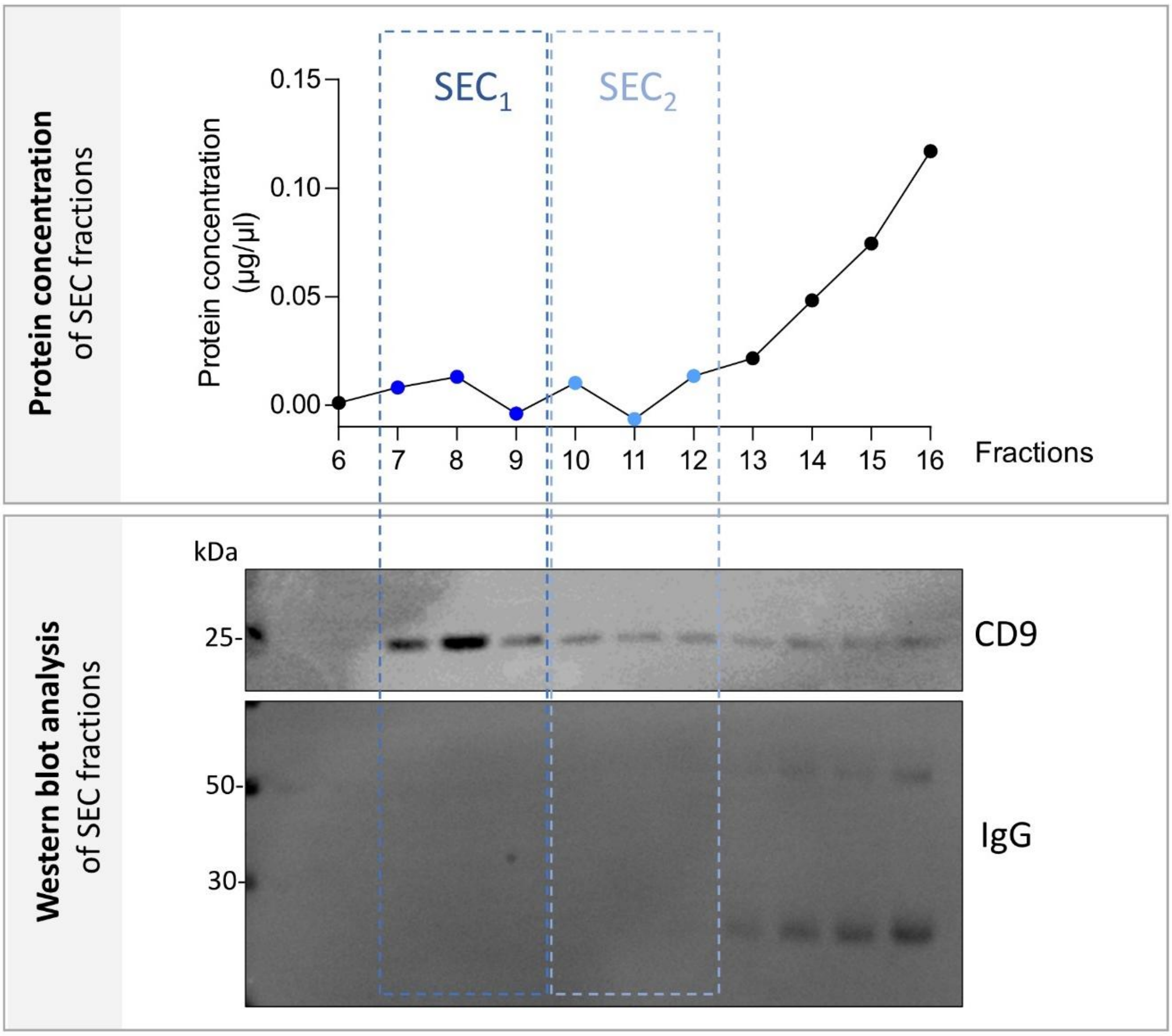

Supplementary Figure 1.

Overview of the SEC-based EV isolation workflow

To obtain EV-enriched fractions, samples eluting prior to the peak in the BCA assay curve are selected. The selected fractions are further analyzed using SDS-PAGE followed by Western Blot (WB) probing for CD9 and IgG. Fractions exhibiting high CD9 and low IgG signals are combined (typically fractions between 7 to 10) and concentrated, yielding sample "SEC_1". The fractions following those used for SEC_1 samples (typically 2-3 fractions around fractions 10 to 12) are further combined, concentrated, and referred to as samples "SEC_2".

Images of whole blots can be found in the Supporting material.

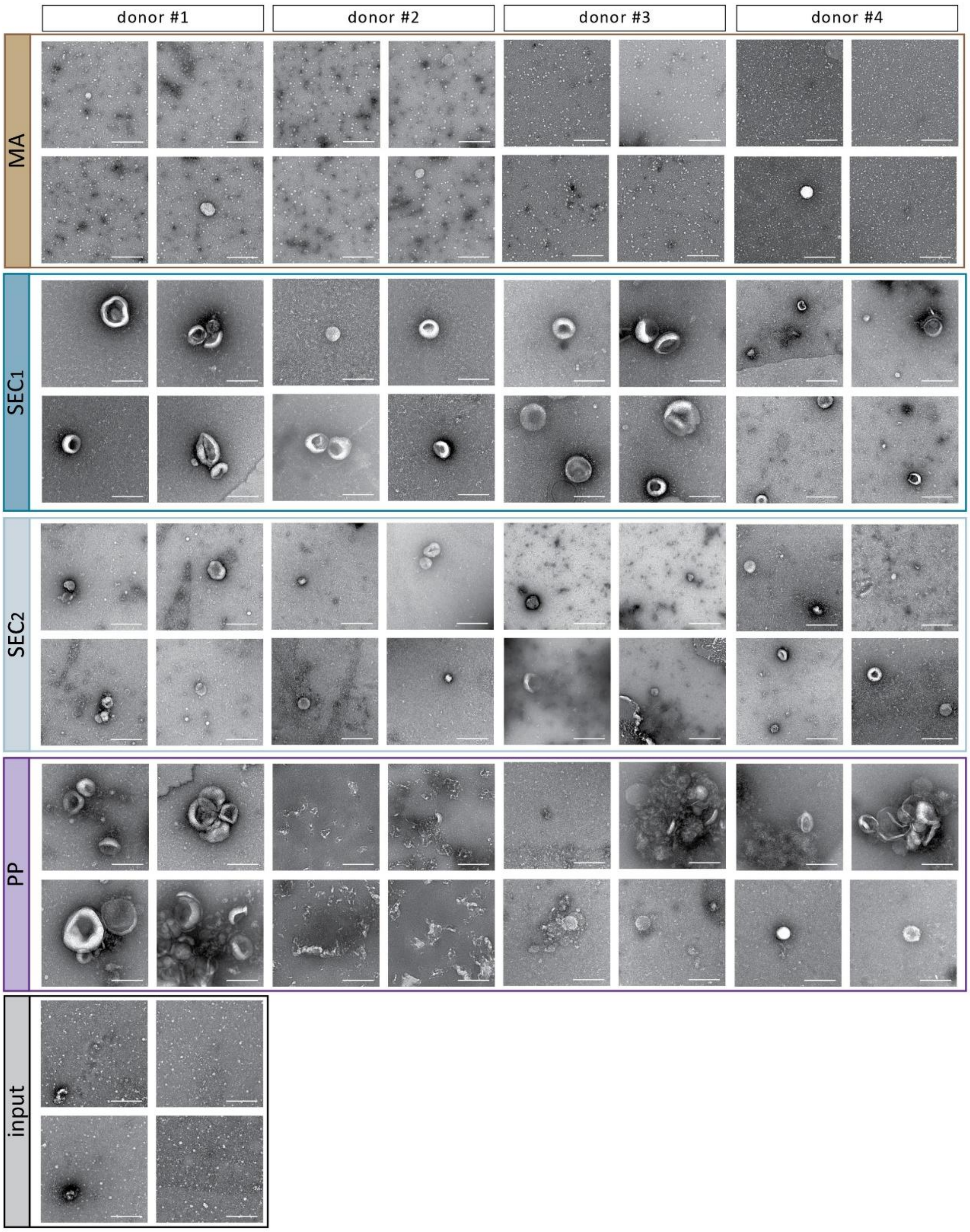

Supplementary Figure 2.

Representative TEM images of salivary EVs from 4 donors isolated with 3 different preparative methods

Scale bar: 200 nm

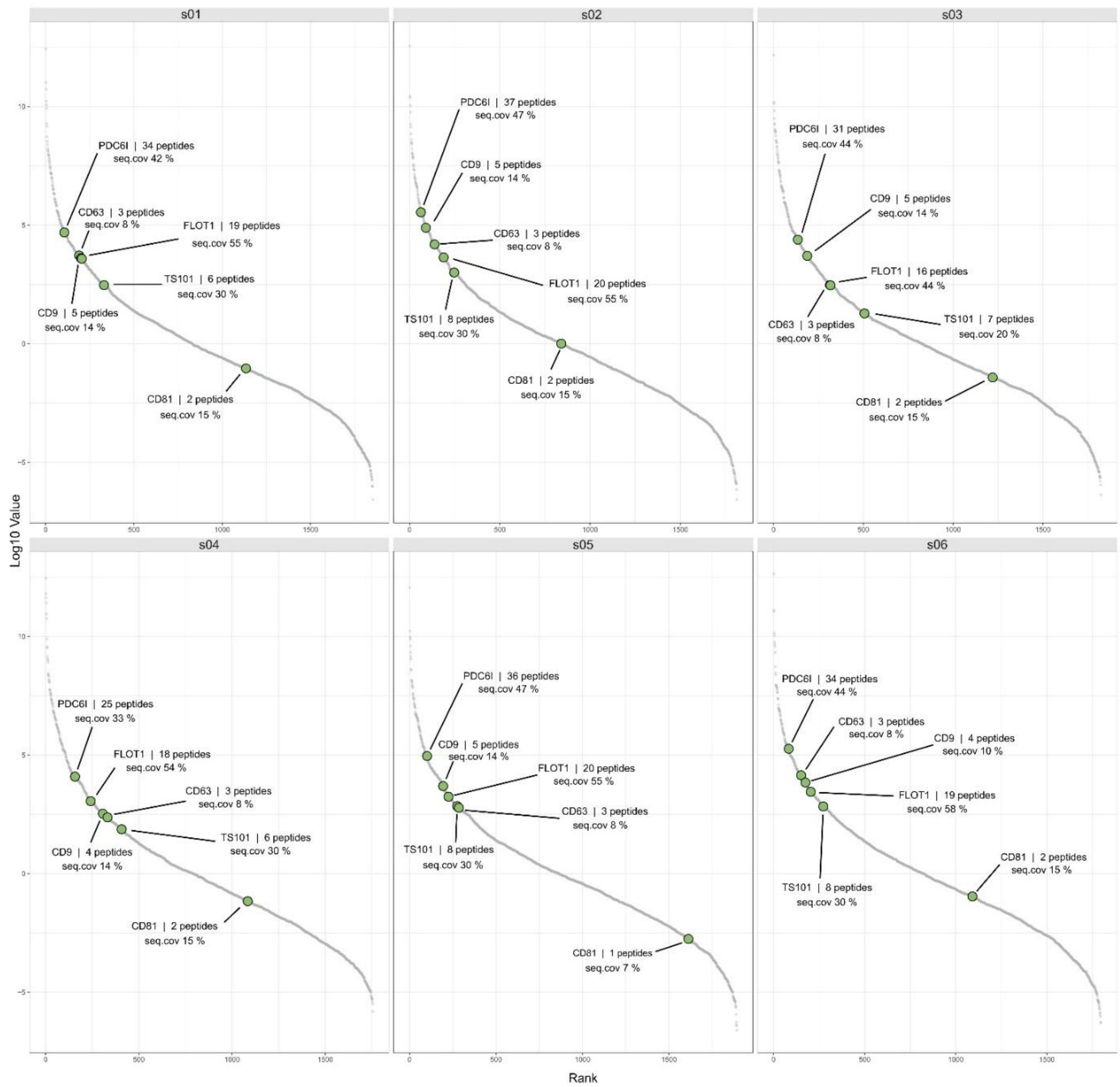

Supplementary Figure 3.

Proteomics analysis of salivary EV samples of 6 donors.

Protein abundance rank plots with positive EV markers (highlighted in green) of salivary EV samples prepared with SEC method from samples of 6 donors.

All selected positive EV markers (Flot1, PDCD6IP/Alix, CD9, CD63, CD81, TS101/TSG101) are detected in all six samples, presenting similar peptide coverage, and protein abundance profiles across the analyzed samples.

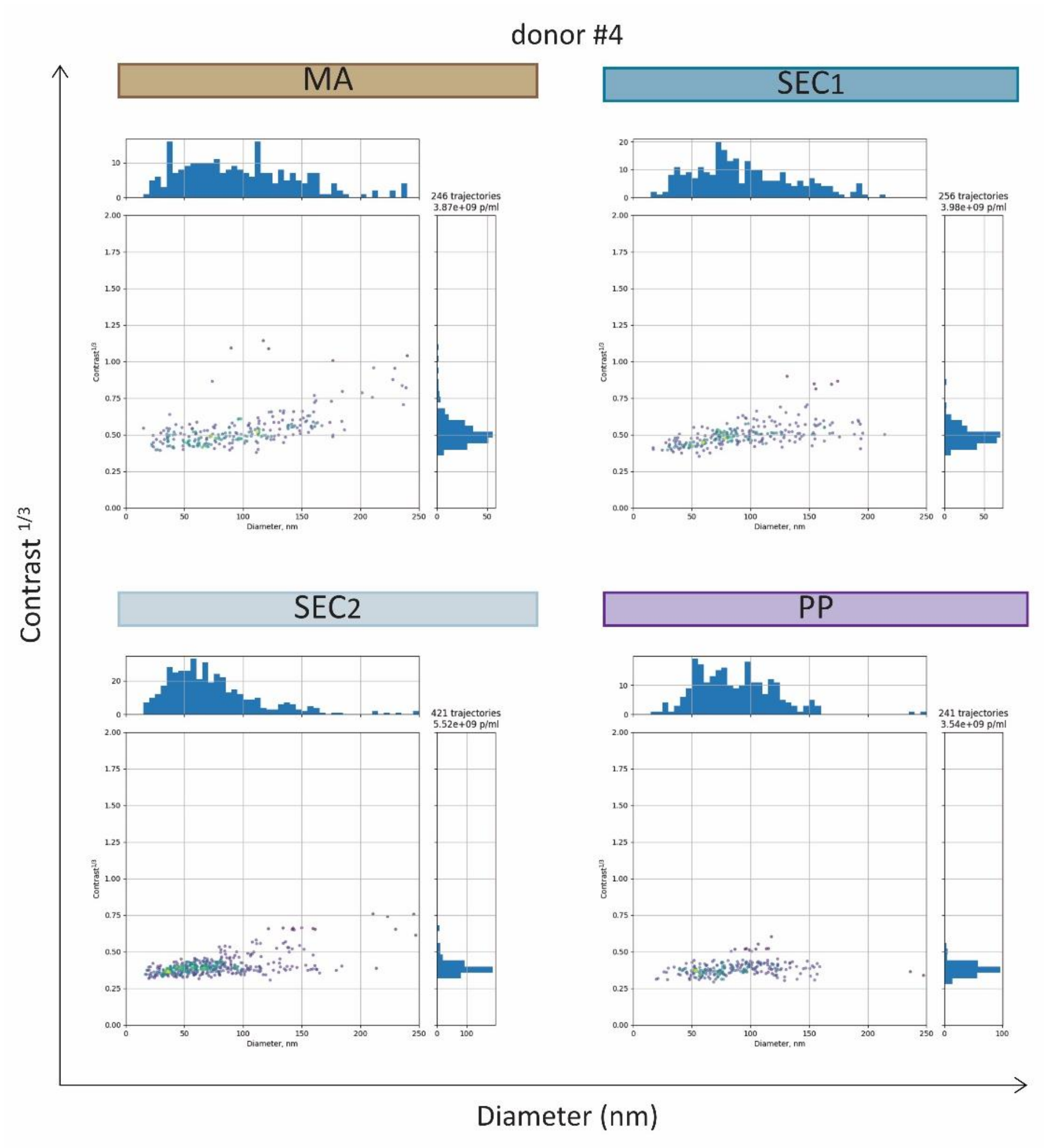

Supplementary Figure 4.

iNTA analysis of salivary EVs.

Representative graphs of salivary EV samples of one donor prepared with different isolation methods. Graphs show the particle diameter plotted against the third root of iSCAT contrast.

Supporting material

Uncropped images of TEM images presented in Fig. 3.

Scale bar: 200 nm

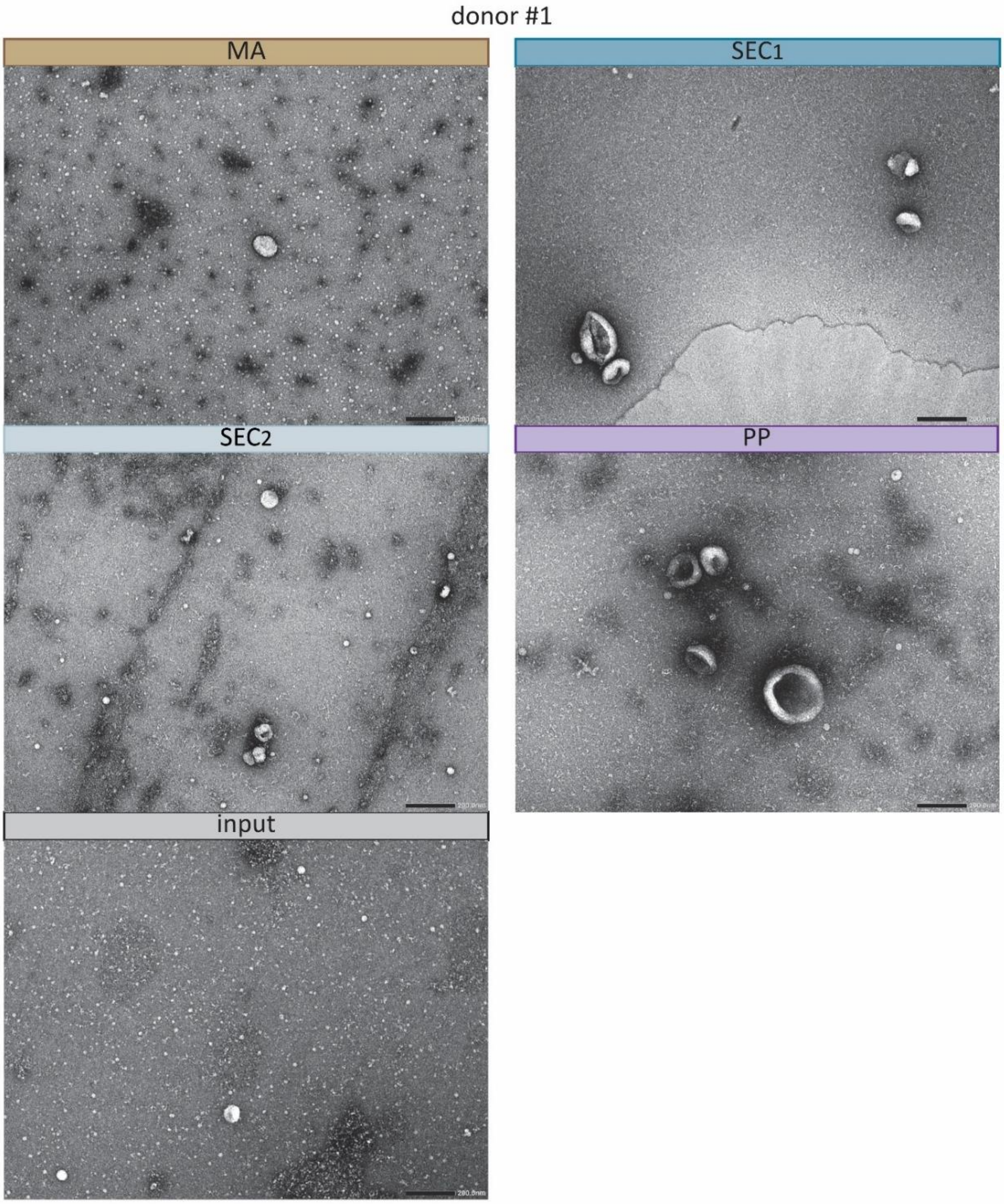

donor #2

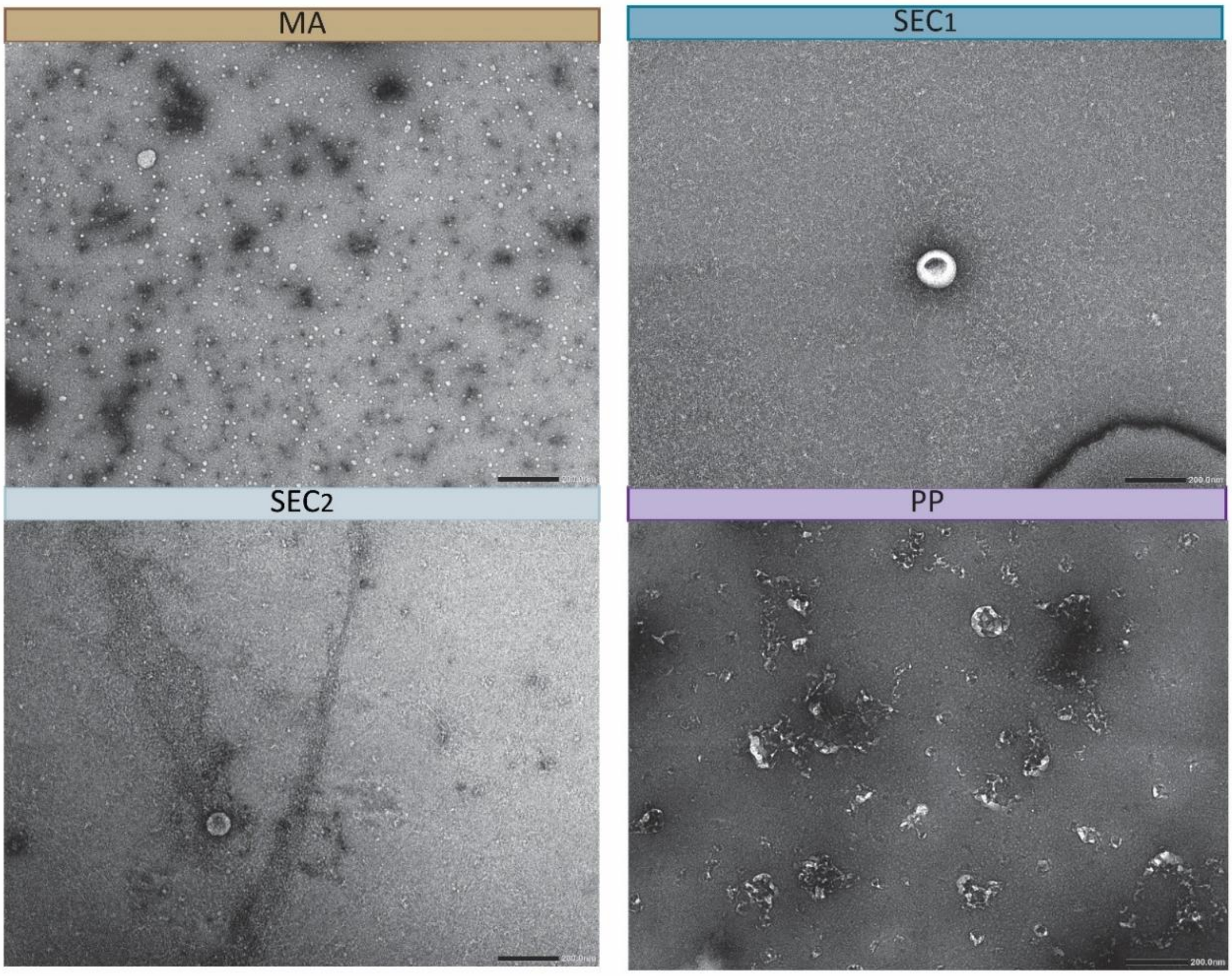

donor #3

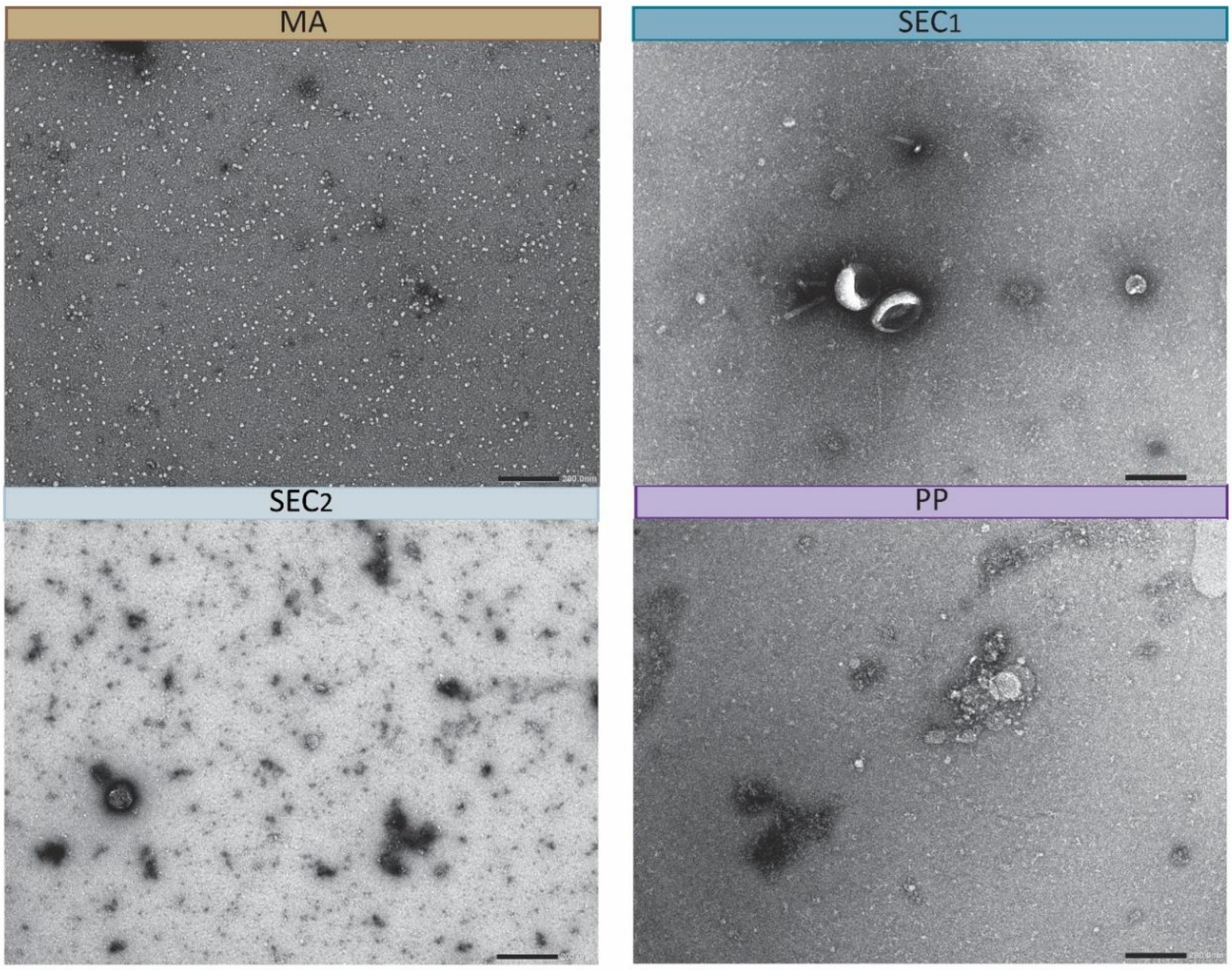

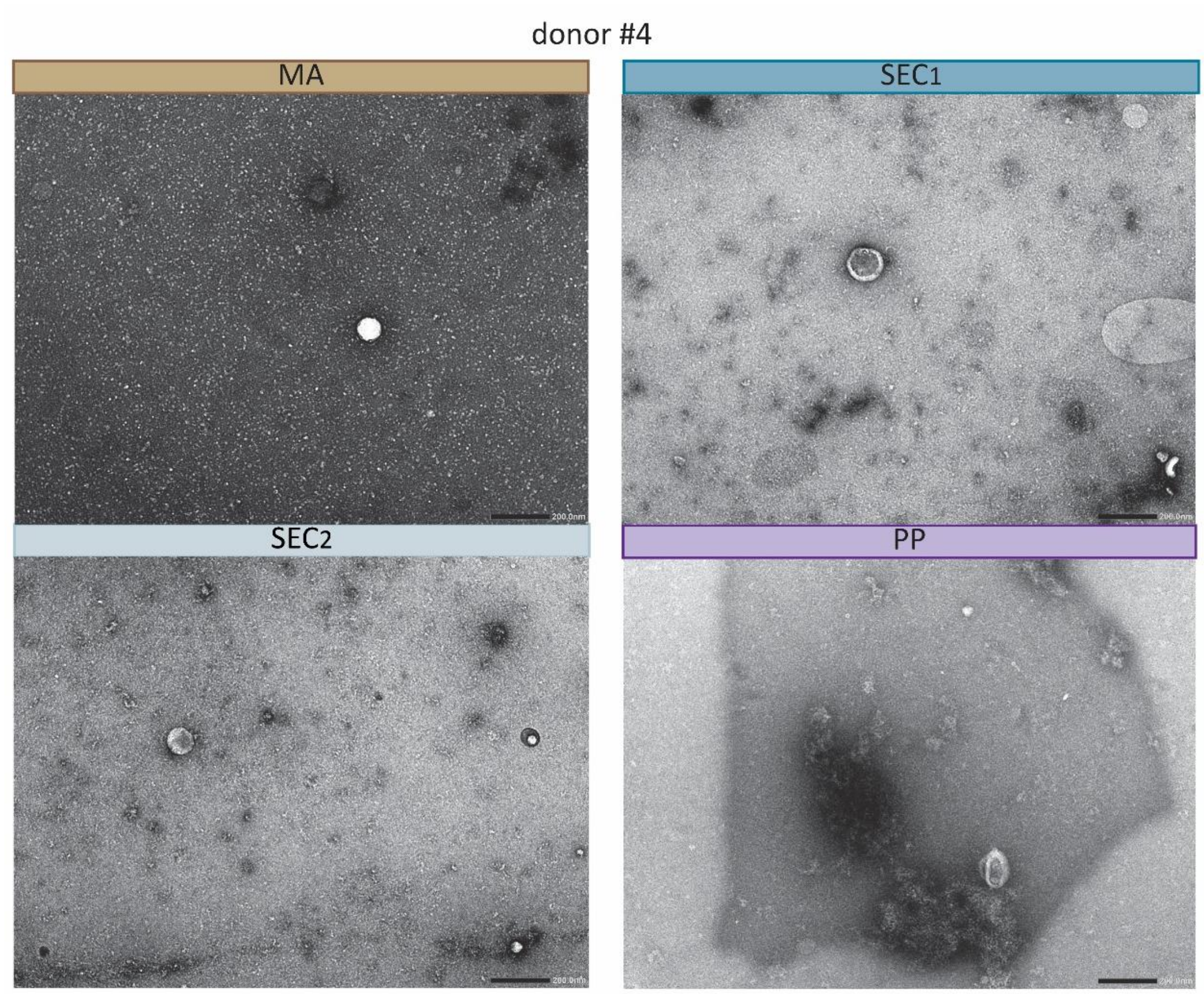

Whole blots and protein gels of images presented in Fig. 4.

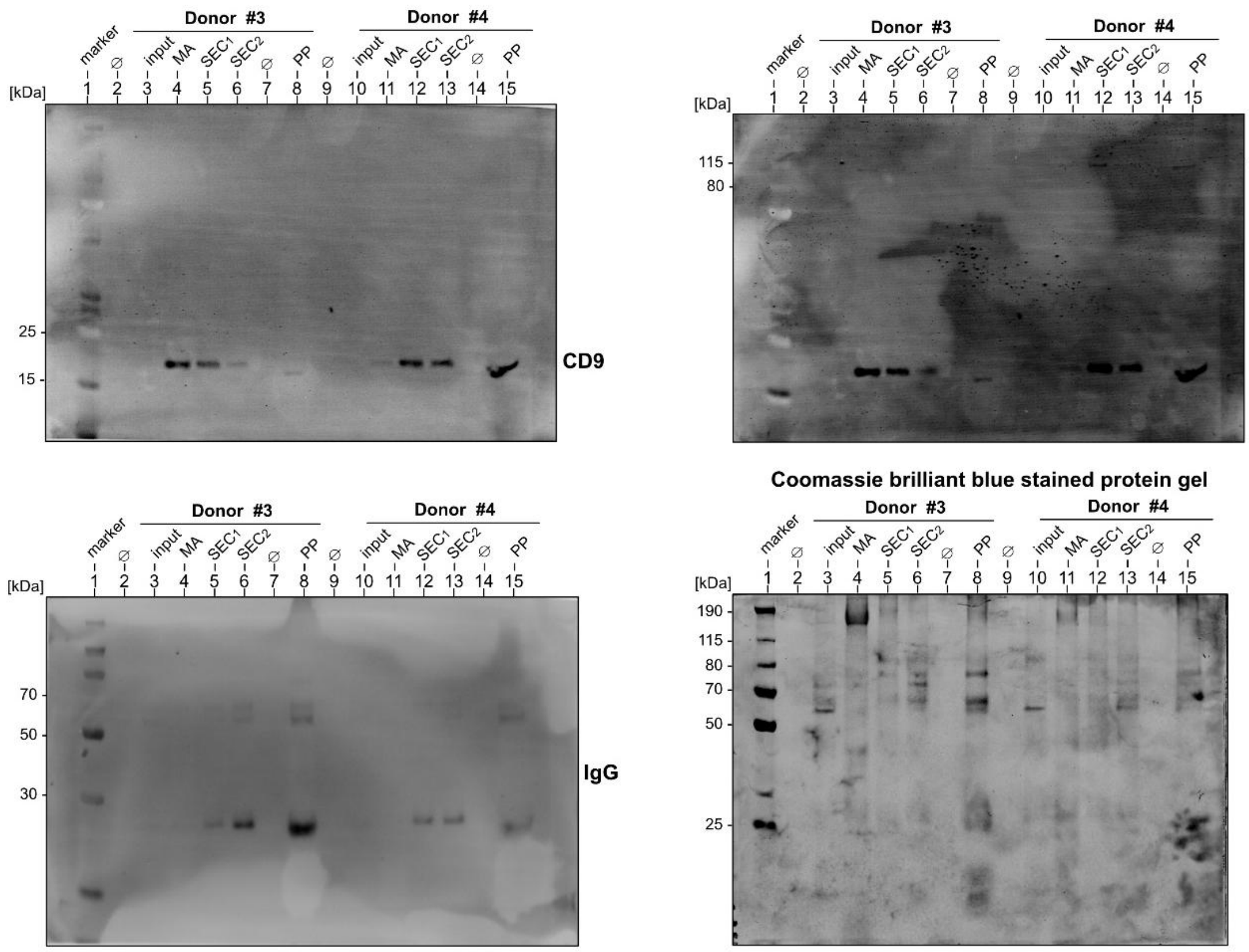

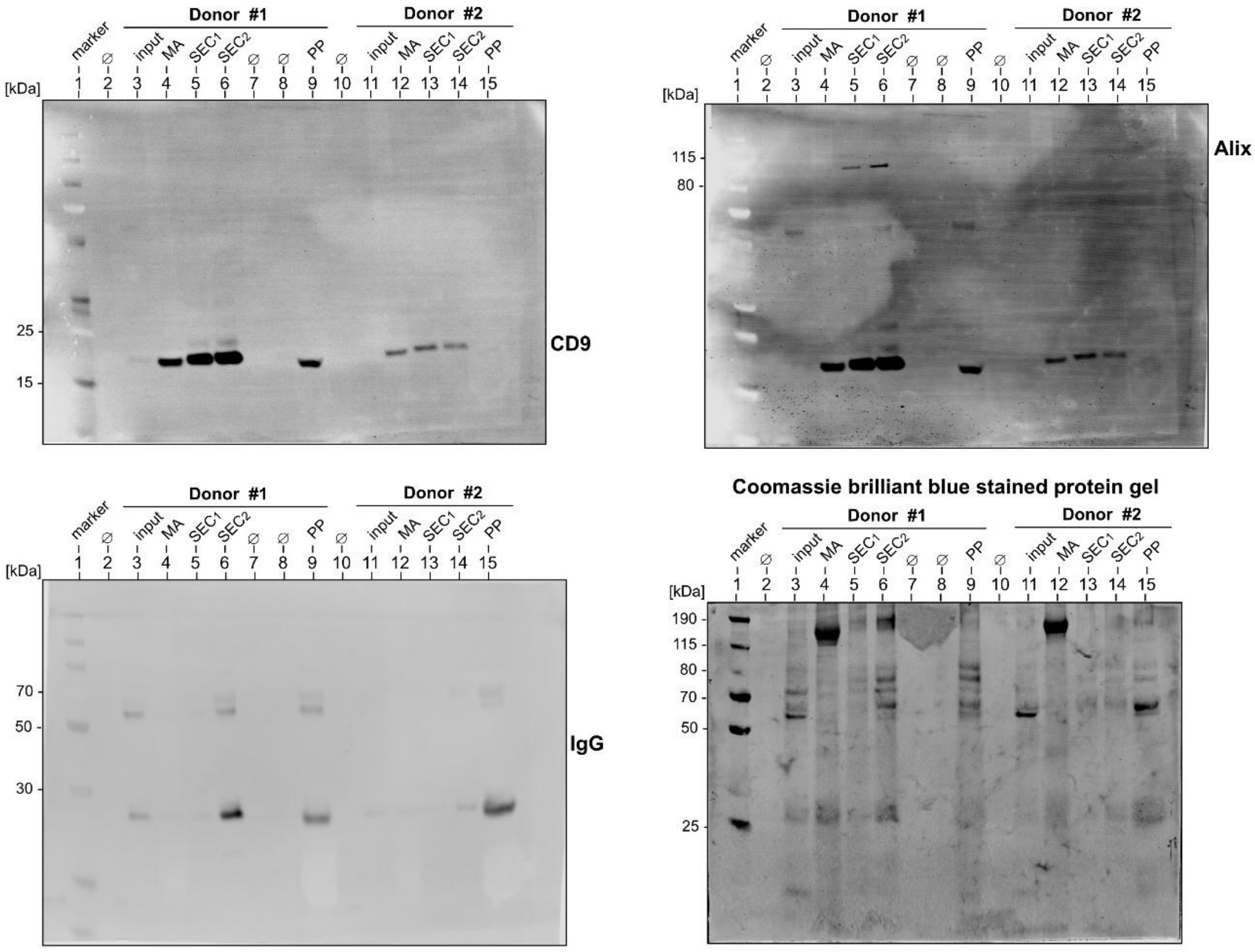

Whole blots and protein gels of images presented in Suppl. Fig. 1.

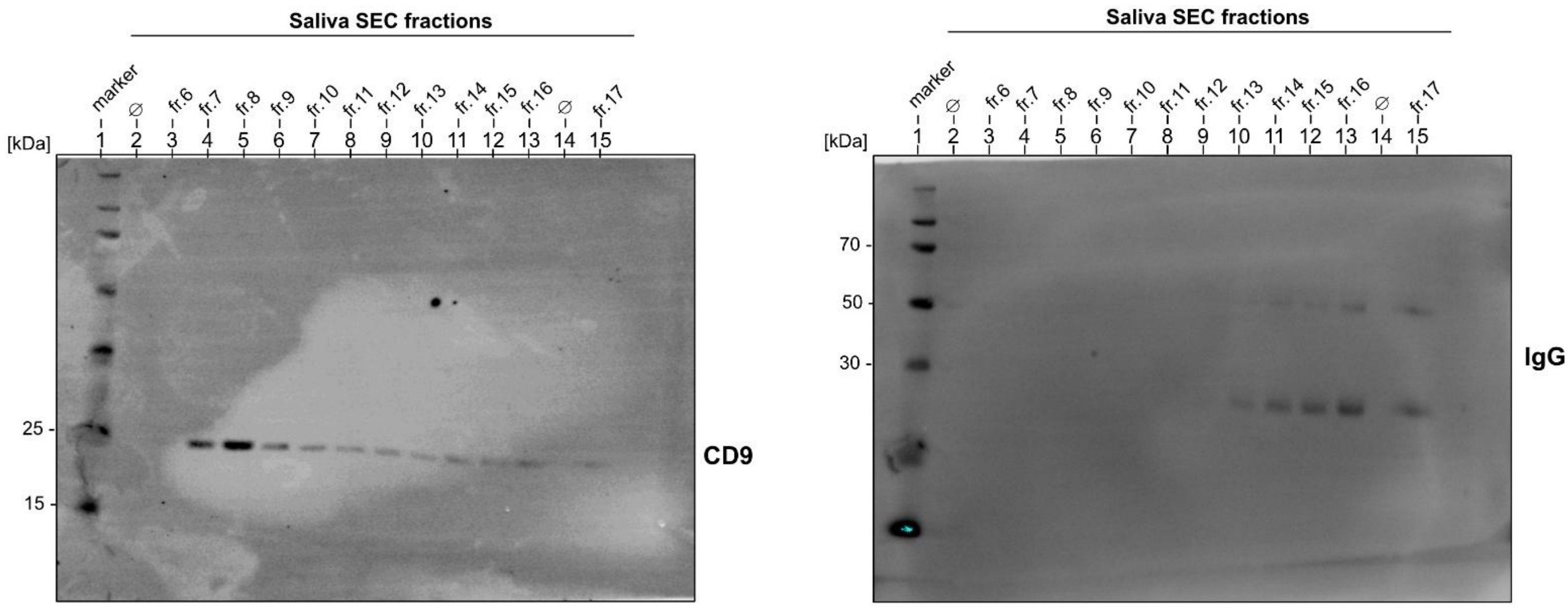

Supplement: Supplementary file 1 [file mmc1.pdf]
